# A closed-loop framework for descriptor-empowered exploration of high-activity Co-based quaternary molten alloy catalysts for methane pyrolysis

**DOI:** 10.1039/d6sc05383a

**Published:** 2026-09-03

**Authors:** Hangwei Liu, Zihao Cheng, Xuxuan Huang, Yangdong He, Wenhan Wang, Wei Yang, Jinwen Li, Zhaoyu Huang, Dong Zhao, Junmei Du, Teli Lin, Seok Ki Kim, Hao Li, Yongliang Tang, Yuanzheng Chen

**Affiliations:** a School of Physical Science and Technology, Southwest Jiaotong University Chengdu Sichuan 610031 China tyl@swjtu.edu.cn cyz@swjtu.edu.cn; b Research Institute of Natural Gas Technology, PetroChina Southwest Oil & Gasfield Company Chengdu 610213 P. R. China; c Advanced Institute for Materials Research (WPI-AIMR), Tohoku University Sendai 980-8577 Japan li.hao.b8@tohoku.ac.jp; d Department of Energy Systems Research, Ajou University Suwon 16499 Republic of Korea

## Abstract

Methane (CH_4_) pyrolysis *via* molten media offers an attractive route to CO_2_-free hydrogen production with high-value carbon as a co-product. However, the rational design of multicomponent molten catalysts remains challenging due to the dynamic and disordered nature of active sites in the molten state. Here, we present a closed-loop framework that integrates potential catalyst exploration by descriptor data mining, CH_4_ pyrolysis testing, and activity mechanism analysis to identify high-activity catalysts for CH_4_ pyrolysis. Using this approach, Bi_70_Ni_18_Cu_6_Co_6_ is identified as a promising catalyst, with experimental validation demonstrating that it can achieve 40.3% CH_4_ conversion at a moderate 1000 °C with a relatively low activation energy (197.3 kJ mol^−1^), outperforming Bi_73_Ni_27_ (33.3%) and Bi_76_Ni_18_Cu_6_ (37.4%) molten media under the same experimental conditions. The enhancement stems from a synergistic multi-solute effect, whereby the co-dissolution of Ni, Cu, and Co in the low-surface-tension Bi solvent increases the density of exposed active sites and promotes direct interaction with CH_4_ molecules. In particular, introducing Co downshifts the LUMO energy level of the CH_4_ molecular orbital (∼1.3 eV), facilitating charge antibonding occupation, weakening C–H bonds, and thereby accelerating CH_4_ dissociation. These findings provide deep insights into Co-based multi-elementary molten catalysts and chart a clear path toward the rational design of high-performance multicomponent molten catalysts for CH_4_ pyrolysis.

## Abstract 2

A closed-loop, descriptor-driven strategy integrates data mining, computation, experiments, and mechanistic analysis to discover high-activity molten alloy catalysts for methane pyrolysis.

## Graphical abstract

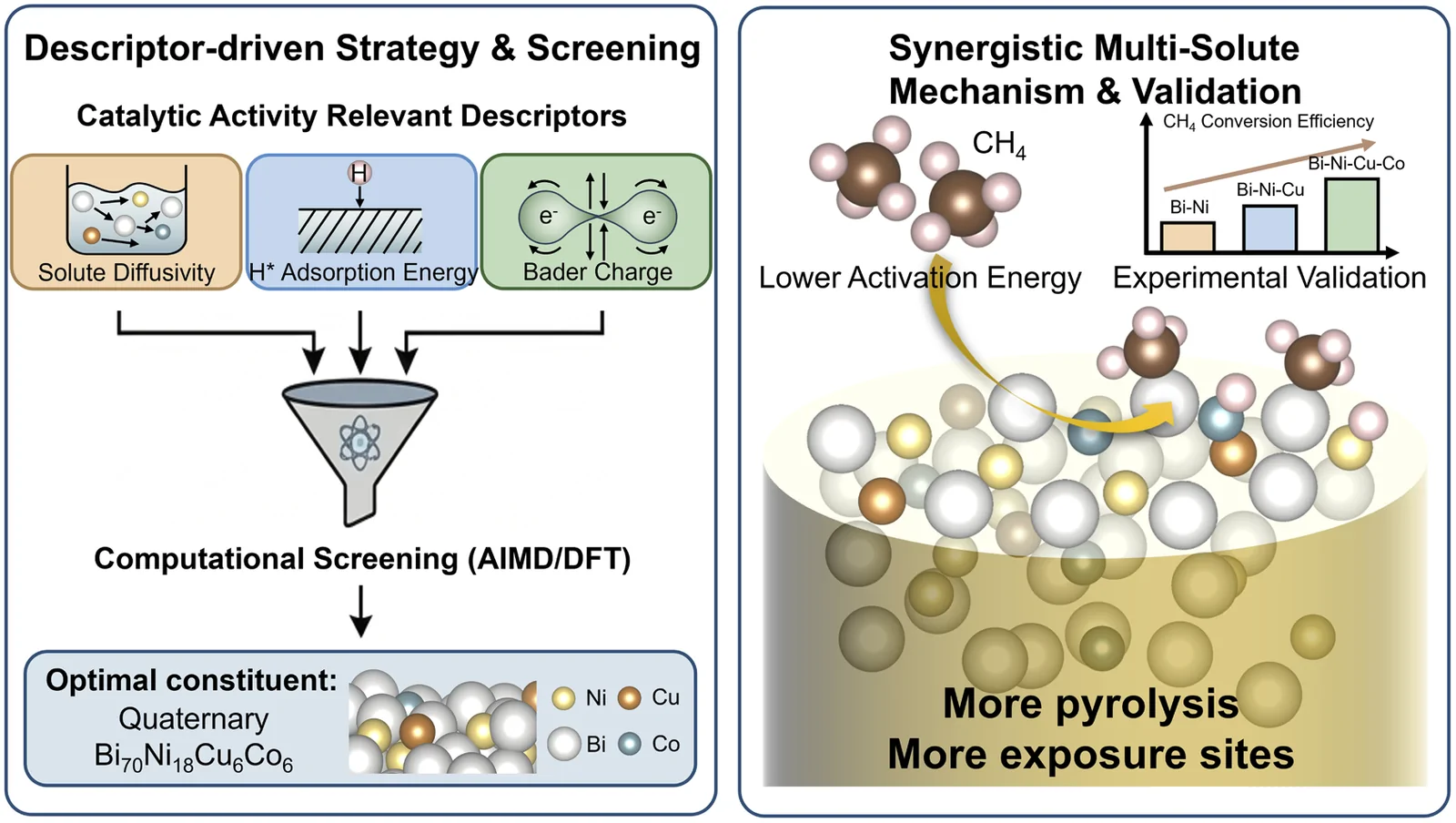

## 1. Introduction

Natural gas, as one of the world's most abundant fossil fuel resources, plays a pivotal role in global energy systems.^1,2^ However, its direct combustion releases substantial carbon dioxide (CO_2_) emissions, contributing negatively to global warming and thus driving scientific efforts toward decarbonization through hydrogen (H_2_) production. While H_2_ represents a clean, efficient energy carrier crucial for the transition to carbon neutrality, conventional methods like steam methane reforming and coal gasification generate significant CO_2_, and water electrolysis remains constrained by high energy costs.^3,4^ Among alternative pathways, CH_4_ pyrolysis has emerged as a promising route for decarbonizing natural gas, producing H_2_ without direct CO_2_ emissions while yielding valuable solid carbon co-products.^5,6^ This process achieves clean energy conversion through controlled thermal decomposition of CH_4_, though uncatalyzed reactions require prohibitively high temperatures (>1000 °C), making catalytic strategies essential for practical implementation.^7^

In the early stage, solid catalysts were used for CH_4_ pyrolysis, which can significantly lower the temperature required for CH_4_ pyrolysis down to 500–800 °C, but carbon produced from CH_4_ pyrolysis often quickly encapsulates solid particles and deactivates catalysts.^6–8^ In recent years, a molten catalyst cracking method has gradually emerged as a promising solution for CH_4_ pyrolysis.^9^ This method employs molten media as both a catalyst and a heat transfer medium to facilitate CH_4_ pyrolysis, which has garnered great attention due to its superior advantages such as high conversion efficiency, easy separation of products, and production of potentially high-value carbon (*i.e*., graphene^10,11^ and diamond^12^). One important advantage is that molten catalysts offer a liquid reaction environment, in which accumulated solid carbon can potentially be readily transported and removed, thus preventing deactivation by coke deposition that happens in solid catalysts.^13^ Recent reports on the catalysis of multi-metal/nonmetal elements mixed in molten media, particularly molten metal alloys, have shown great potential for effective CH_4_ pyrolysis at a moderate temperature. In 2017, Upham *et al.* reported that a 27% Ni–73% Bi molten alloy achieved 95% CH_4_ conversion at 1065 °C.^14^ On the basis of this binary alloy, Chen *et al.* further proposed a high-efficiency ternary molten metal alloy 2.3 wt% Ni–96.4 wt% Bi–1.3 wt% Mo, reaching a hydrogen production efficiency of 4.05 ml g^−1^ min^−1^ even at a low temperature of 800 °C.^15^ These studies demonstrated the superior performance of molten alloys with highly effective catalytic activity for CH_4_ pyrolysis.

Despite the demonstrated efficacy of molten alloys in CH_4_ pyrolysis, their rational design faces fundamental challenges arising from the vast compositional space and dynamic disorder of atomic arrangements under extreme conditions. The emergence of data-driven approaches has enabled descriptor-guided design methodologies that systematically correlate structural features with catalytic performance.^16–22^ Early studies identified charge transfer behavior as a critical descriptor, revealing that solute atoms with less negative charge enhance CH_4_ activation—a principle successfully applied in designing Zn–Bi, Cu–Bi, and Se–Ni–Bi systems.^23–25^ Recent advances have expanded the descriptor toolbox to include hydrogen binding energy (reflecting CH_3_–H affinity) and solute diffusivity (governing active-site accessibility), enabling the computational discovery of Bi–Ni–Cu and Bi–Ni–Mn ternary alloys that outperform their Bi–Ni binary counterparts, with experimental validation confirming their superior catalytic performance.^17^ This physical ground can enable the translation of these activity descriptors of CH_4_ pyrolysis into actionable design principles for the rational design of high-performance molten media,^16^ empowering a descriptor-driven molten catalyst design paradigm for CH_4_ pyrolysis.

In this work, we establish a closed-loop framework to identify high-activity catalysts for CH_4_ pyrolysis, integrating potential catalyst exploration by descriptor data mining, CH_4_ pyrolysis testing, and activity mechanism analysis. The framework begins with catalyst screening *via* data mining using the “Descriptors” module, derived from our AI-empowered digital catalysis methane pyrolysis agentic platform (DigMethpy, https://www.digmethpy.org)^26^ and *ab initio* molecular dynamics (AIMD) simulations combined with density functional theory (DFT) calculations. Experimental tests and advanced characterization validate performance, while multi-scale simulation and analysis refine the understanding of catalytic mechanisms. The findings and data are then incorporated into the DigMethpy platform, creating a feedback loop that accelerates future discoveries. Through this approach, Bi_70_Ni_18_Cu_6_Co_6_ is identified as a promising catalyst, and experimental validation demonstrates that it can achieve 40.3% CH_4_ conversion at a moderate 1000 °C with a relatively low activation energy (197.3 kJ mol^−1^), outperforming Bi_73_Ni_27_ and Bi_76_Ni_18_Cu_6_ molten media under the same experimental conditions. We elucidated the underlying mechanism of multicomponent molten metals and revealed a synergistic catalytic effect of multiple solutes. This case showcases the strength of our integrated, loop-based discovery strategy for the rational design of high-performance systems tailored for CH_4_ pyrolysis.

## 2. Experimental section

### 2.1 Computational details

The DFT calculations were conducted using a plane-wave-based Vienna *Ab initio* Simulation Package (VASP).^27,28^ The electron–ion interactions are described using the Projector Augmented Wave (PAW) potential,^29^ and the Perdew–Burke–Ernzerhof (PBE) functional within the Generalized Gradient Approximation (GGA) is adopted for exchange–correlation energy,^30^ achieving a balance between accuracy and computational cost. To accurately account for long-range interactions between metal atoms, the dispersion correction method (DFT-D3) proposed by Grimme is incorporated,^31^ effectively compensating for the deficiencies of conventional GGA-PBE in describing van der Waals forces, thus ensuring the accuracy of calculations involving weak adsorption and surface reaction barriers. For parameter settings, the plane-wave basis set cutoff energy is set to 600 eV. In the model construction, we adopt a supercell with dimensions of 15 Å × 15 Å × 30 Å containing 100–200 metal atoms. The initial arrangement of atoms is randomized to simulate the amorphous surface structure formed after melt solidification. To better reflect the surface structural characteristics of molten metal bubbles, the atomic positions within the bottom 7 Å of the model are fixed to maintain the stability of the bulk phase structure, while the top-layer atoms are fully relaxed during structural optimization. A vacuum layer of 20 Å is applied to eliminate interactions between the surface and its periodic images under periodic boundary conditions. The Brillouin zone is sampled with a Monkhorst–Pack 2 × 2 × 1 *k*-point mesh, which is sufficient to ensure energy and force convergence by our convergence tests.^32^ To investigate the structural evolution and atomic diffusion behavior of high-temperature molten metal bubbles, *ab initio* molecular dynamics (AIMD) simulations are performed. The canonical ensemble (NVT) is established using a Nose–Hoover thermostat^33,34^ with a simulation temperature of 1500 K. The initial simulation time is 10 ps with a time step of 1 fs.

The mean square displacement is calculated from the trajectory data to obtain the diffusion coefficients of different metal elements, which are then used to analyze the dynamic reconstruction of active sites on the alloy surface during high-temperature reactions. The self-diffusion coefficient (diffusivity, *D*) of the metal was calculated from the mean-squared displacements (MSD) using the following equation:^35^1
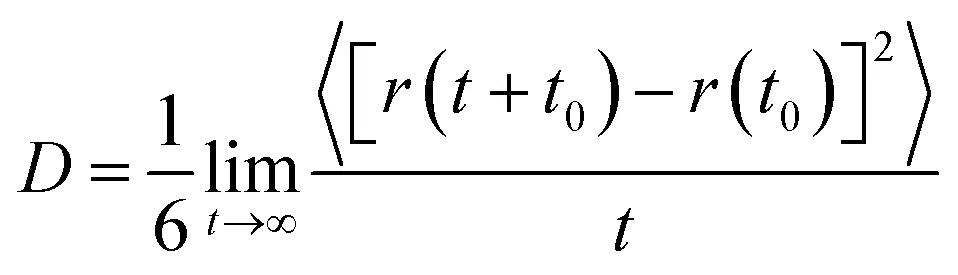
where *r*(*t*) is the MSD at time *t*. The self-diffusion coefficient of the solute in ternary metal alloys (*D*_Bi–Ni–Cu–X,solute_, where X represents the solute metals of different elements added) was mole-averaged using the following equation:2
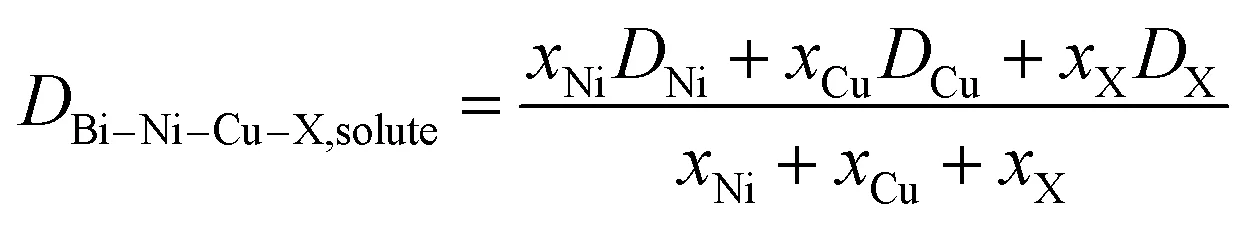
where *x*_*i*_ represents the mole fraction and *D*_*i*_ represents the self-diffusion coefficient of the metal species *i*. The adsorption energy of hydrogen atoms on the alloy surface (Δ*E*_H*_) was calculated using the following equation:^36^

3 Δ*E*_H*_ = *E*_H*_ − *E*_slab_ − *E*_H_2__/2

where *E*_H*_ is the total energy after H* adsorption, *E*_slab_ is the total energy of the clean surface, and *E*_H_2__ is the energy of a gas-phase hydrogen molecule. The atomic charges and the associated charge-transfer magnitudes were determined using Bader charge analysis.^37^ For reaction kinetics simulations, the Climbing Image Nudged Elastic Band method is employed to determine the dissociation pathway of methane molecules on the surface and the corresponding activation barriers. To ensure reliable transition state identification, vibrational frequency analysis is performed for each candidate structure, retaining only those with a single imaginary frequency as valid TS structures.^38^ Implicit solvation calculations for the Co-added CH_4_ molecule model are conducted using the GLSSA13 solvent model,^39^ implemented in VASP *via* the VASPsol extension.^40,41^ The pCOHP analysis was performed using the LOBSTER 4.1.0 package.^42,43^

### 2.2 Materials and reaction test

The metallic raw materials used in the experiments include high-purity (99.9%) bismuth (Bi) granules, nickel (Ni) spheres, copper (Cu) granules, and cobalt (Co) flakes, all purchased from the China Iron & Steel Research Institute and used without further purification. The alloy preparation process is conducted in a specially designed bubble column reactor. Prior to the experiment, the metal particles are weighed according to the designed molar ratio, mixed uniformly, and loaded into the molten pool region of the reactor. A metal catalyst layer approximately 20 cm in height is loaded into the ceramic tube. The reaction gas enters from the inlet end, passes through the high-temperature catalyst bed where methane cracking occurs, and exits from the outlet end. To prevent oxidation of the metals at high temperature, 60 sccm of 99.99% high-purity argon gas is introduced before heating to completely purge the internal atmosphere of the reactor. The temperature is then increased to 1400 °C at a rate of 10 °C min^−1^ under atmospheric pressure to ensure complete melting and uniform mixing of all metal components. During the high-temperature holding stage, argon gas is continuously supplied to maintain an inert atmosphere. The molten metal catalyst is subsequently subjected to reduction at 1000 °C for 3 h under a continuous flow of 30 sccm H_2_ and 10 sccm Ar to ensure complete activation of the alloy surface. After the melt is formed, the temperature is slowly reduced to 1000 °C, at which point the atmosphere is switched to a mixed gas of 50% CH_4_ + 50% Ar at a total flow rate of 40 sccm to initiate the methane cracking reaction. During the reaction, the gas is bubbled directly into the molten metal through an inlet pipe at the bottom of the reactor, maximizing the gas–liquid interfacial contact area and thereby improving reaction efficiency. The effluent gas is analyzed qualitatively and quantitatively using gas chromatography.

## 3. Results and discussion

### 3.1 Workflow of the closed-loop framework

The workflow employed in this study (Fig. 1) consists of three integrated stages: catalyst exploration, synthesis and CH_4_ pyrolysis testing, and activity analysis of the catalyst, connected through a closed feedback loop:

**Fig. 1:**
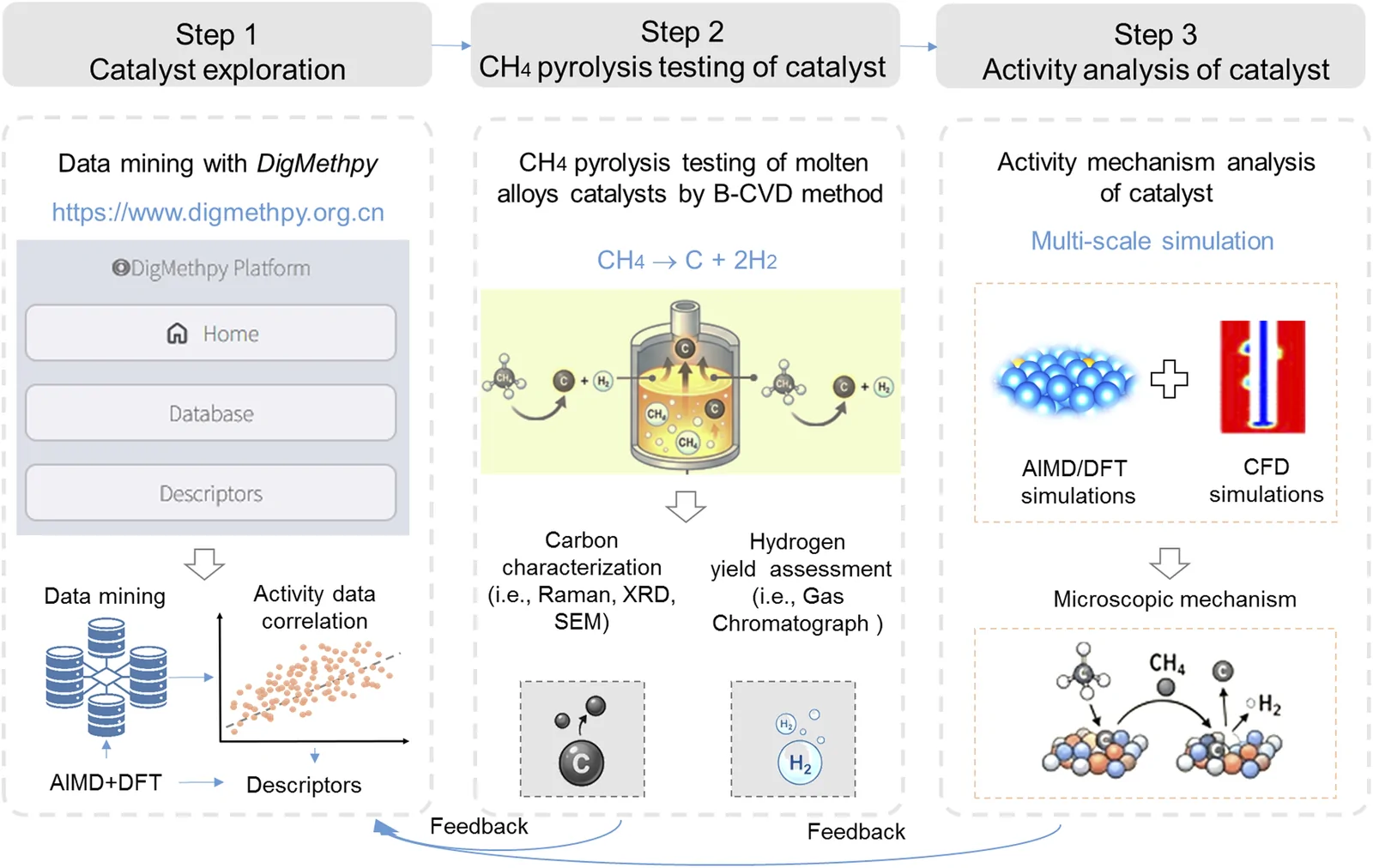
Schematic illustration of a closed-loop framework integrating descriptor-driven catalyst exploration, experimental validation, theoretical mechanism analysis, and iterative feedback optimization of the DigMethpy platform and descriptor model.

• Step 1 – catalyst exploration: this process begins with data mining using the “descriptor” module within the DigMethpy platform to identify the activity descriptors of molten metal systems for CH_4_ pyrolysis. Then, according to the computational descriptor data from AIMD simulations and DFT calculations, it performs the screening of Bi–Ni-based alloy catalysts.

• Step 2 – CH_4_ pyrolysis testing of the catalyst: the selected alloy catalyst is synthesized and evaluated for CH_4_ pyrolysis under high-temperature molten conditions. Subsequently, the pyrolysis product of the catalyst is collected and characterized by advanced techniques, including Raman, XRD, and SEM, in the experimental stage.

• Step 3 – activity analysis of the catalyst: the catalytic mechanism of the catalyst was analyzed and refined. The findings and related data are then incorporated into the DigMethpy database, creating a feedback loop to refine the descriptor–activity relationships and accelerate more precise design in the future.

For closing the feedback loop, the experimental and theoretical data validate the results from catalyst exploration integrated with data mining within DigMethpy and activity descriptor evaluation. The performance of the tested Bi–Ni-based catalyst is compared with the entries in the “Database” and subsequently integrated into this module to expand and enrich the platform. Through this iterative feedback process, discrepancies between predicted and experimentally measured catalytic performance can be identified and used to adjust descriptor selection criteria and improve the predictive capability of the descriptor model. This closed-loop framework will contribute to guiding future refinements, enabling more accurate identification of high-activity catalysts in data mining efforts, and accelerating the discovery of promising molten alloy catalysts for CH_4_ pyrolysis.

### 3.2 Data mining for activity descriptors and selection of the Bi–Ni–Cu system as a case study to design quaternary alloys for CH_4_ pyrolysis

The “Descriptors” module within DigMethpy is designed to evaluate the catalysis activity descriptors of molten catalysts for CH_4_ pyrolysis. This module is derived from the DigMethpy platform that contains more than 40 000 curated data points from over 500 literature sources together with computationally generated records, spanning molten metals, alloys, salts, and their mixed multicomponent systems relevant to methane pyrolysis. To gain insights into the activity of molten catalysts, we analyzed the key performance metrics within the DigMethpy database (Fig. S1 and S2). The literature understanding and knowledge extraction functions were implemented using Python code and large language models and deployed in DigMethpy. The characteristics that include the solute Bader charge, the solute diffusion coefficient, and the H* adsorption energy on the solute metal surface have been proven to positively correlate with the catalytic activity of molten metal systems for CH_4_ pyrolysis. These characteristic relationships align with prior experimental observations (*i.e.*, Bi-based molten catalysts)^14^ and their potential predictive capability by acting as activity descriptors was partly supported.^17^ This systematic mining enables the use of these parameters as effective descriptors to design molten catalysts for CH_4_ pyrolysis. More details on the data mining and analysis for identifying descriptors are given in our work^26^ and the SI.

The selected descriptors were not only chosen based on statistical correlations but also according to their physical relevance to CH_4_ pyrolysis catalysis. The solute diffusion coefficient describes the dynamic mobility of metal species within the molten alloy, which determines the accessibility and regeneration of catalytic active sites under reaction conditions. A suitable diffusion behavior can facilitate continuous interaction between methane molecules and catalytic components. The H* adsorption energy reflects the adsorption–desorption balance of hydrogen intermediates. Moderate H* binding strength is essential for efficient hydrogen evolution while preventing excessive hydrogen accumulation on the catalytic surface. In addition, Bader charge provides an electronic descriptor describing charge redistribution between alloy components and reaction intermediates, which is closely related to methane C–H bond activation. Together, these descriptors establish a multiscale descriptor framework linking atomic-level properties with catalytic performance. The diffusion descriptor represents the dynamic structural characteristics of molten catalysts, while H* adsorption energy and Bader charge describe reaction intermediate regulation and electronic activation processes, respectively. Their combination enables a more comprehensive evaluation of molten alloy catalysts beyond conventional single-property screening.

With this descriptor insight, we considered these three characteristics as crucial descriptors to screen quaternary molten media for CH_4_ pyrolysis (Fig. 2a). Given that Bi serves as an ideal solvent and Ni/Cu exhibits strong H-binding energy, we extended the Bi–Ni–Cu system to quaternary alloy systems by introducing additional solute metals alongside Ni/Cu. Referring to the molar ratios of typical binary Bi_73_Ni_27_ and ternary Bi_68_Ni_27_Cu_5_ molten alloy systems,^14,17^ we adopted the Bi_70_Ni_18_Cu_6_X_6_ (X denotes the solute metal from transition metals and alkali metals) configuration as a quaternary molten alloy model (Fig. S3). This model ensures that the molar ratio of solute metals (Ni, Cu, and X) is close to the solute/solvent ratio of 1 : 2.5 in the experimentally confirmed high-active binary/ternary molten alloy catalysts (*i.e.*, Bi_73_Ni_27_ and Bi_68_Ni_27_Cu_5_) for CH_4_ pyrolysis. Based on the Bi_70_Ni_18_Cu_6_X_6_ quaternary alloy model, we simulated 20 molten alloy systems with different solute metals X (X = Ca, Sc, Zr, Ti, *et al.*) by AIMD simulations at 1500 K for 10 ps (Fig. S4). After these models reached thermodynamic equilibrium, a mean square Bader charge of solute metals X was analyzed and estimated, as shown in Fig. 2b and Table S1. Note that the absolute values of Bader charge can vary depending on the concentration and location of X metals in the real molten alloy, and thus we only considered their relative tendency of charge transfer rather than the absolute value. For the solute metals X, the Bader charge becomes more negative from group 2 to group 9, but less negative from group 9 to group 12. For most alkali metals, they have extremely low boiling points (600–800 °C), so we only estimated the Bader charge of Ca and Sr due to their relatively high boiling points (>1000 °C). The results show that the positive Bader charges of Ca and Sr are very large. Based on the correlation between charge state and catalytic performance, where lower negative charge corresponds to higher activity for CH_4_ pyrolysis, these two alkali metals do not enhance catalytic activity. In comparison, only transition metals including Mn, Mo, Tc, Fe, Co, Pd, Ag, Cd, and Zn are identified as potential candidates.

**Fig. 2:**
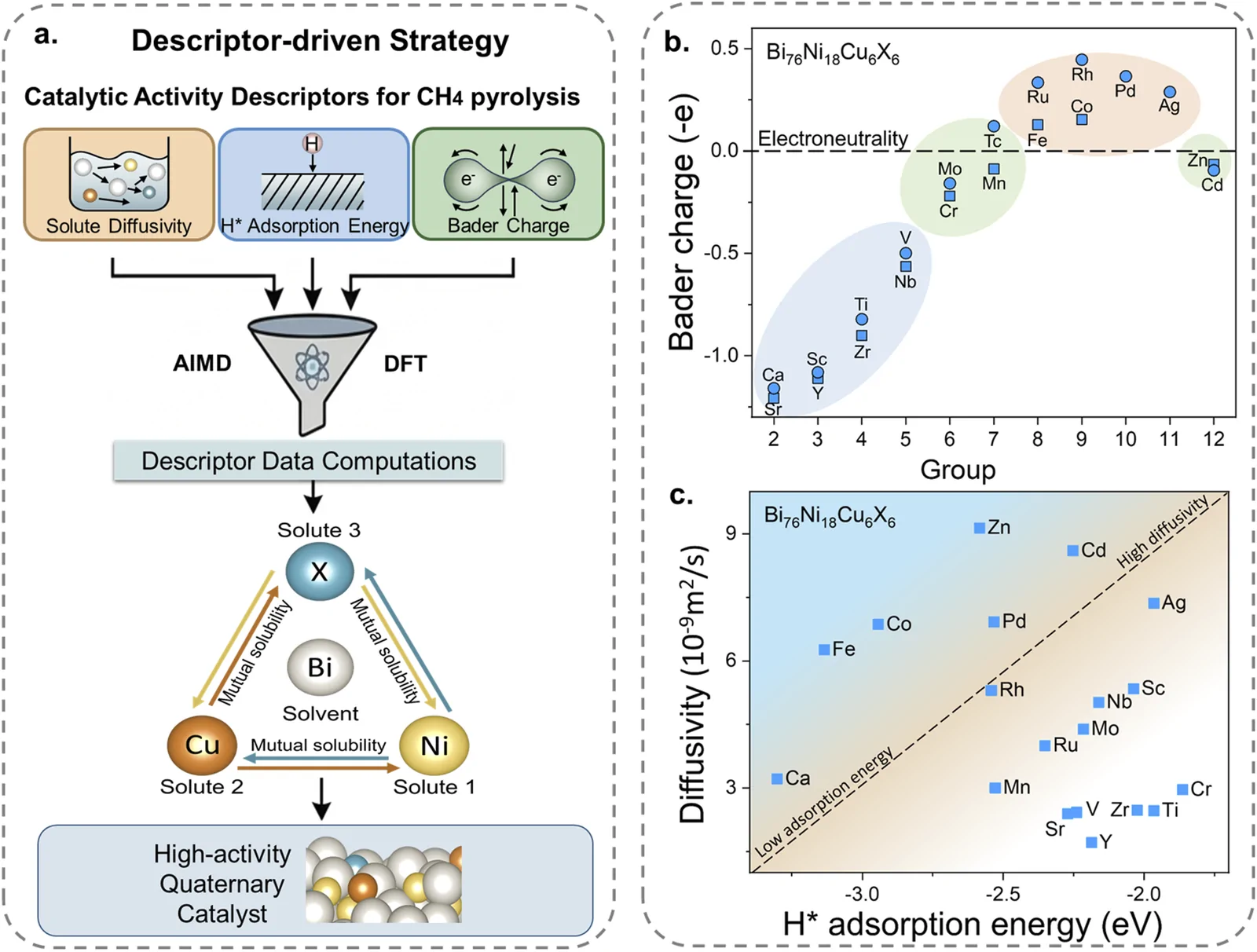
Descriptor-driven catalyst design strategy and descriptor data computations for CH_4_ pyrolysis. (a) Schematic illustration of the descriptor-driven strategy to design quaternary molten alloy catalysts for CH_4_ pyrolysis. (b) Bader charge analysis of solute X metal in Bi_70_Ni_18_Cu_6_X_6_ with reference to the group number of the X element. (c) The H* adsorption energy and diffusion coefficient of solute X metal in various molten alloy Bi_70_Ni_18_Cu_6_X_6_ systems.

Moreover, we further estimated the diffusivity coefficients of solute X metals by MSD calculations in the diffusive regime and computed the H* adsorption energy based on the adsorption surface model incorporating H atoms at the surface X sites *via* DFT calculations (Table S2). Fig. 2c illustrates the partial diffusion coefficients and H* adsorption energies of various X metals in the Bi_70_Ni_18_Cu_6_X_6_ systems. The results show that some solute X metals such as Ca, Fe, Co, Zn, Pd, Rh, and Mn have relatively small negative H adsorption energies, demonstrating strong H* adsorption ability, in which solute metals Fe, Zn, Pd, and Co exhibit high diffusivity with good solubility in Bi–Ni–Cu molten media.

Through the comprehensive assessment of three descriptor factors, Fe, Zn, Pd, and Co emerge as optimal X components. Despite the favorable performance of Pd and Zn in screening, they are excluded from the list of candidates because Pd, as a noble metal, is expensive and Zn has a propensity to vaporize under reaction conditions (>1000 °C).^44,45^

### 3.3 Experimental validation of catalysts for CH_4_ pyrolysis

Based on the above screening results, the experimental validation was subsequently carried out using the established testing platform (Fig. S5). Although Fe and Co can both be selected as the optimal solute metals to further expand the Bi–Ni–Cu molten alloy system, Fe shows low solubility in the Bi–Ni–Cu molten system in practical experiments (Fig. S6), so we only considered Co for further catalytic tests. The tests of CH_4_ pyrolysis were performed by employing a molten bubble CVD (B-CVD) method in a bubble column reactor under high-temperature reduction conditions, as shown in Fig. 3a. To ensure the complete melting of Co and its mutual solubility with other metals, the temperature of the alloy catalyst Bi–Ni–Cu–Co sample needs to be raised to around 1400 °C first and then gradually cooled to 1100–1000 °C for the CH_4_ pyrolysis test.

**Fig. 3:**
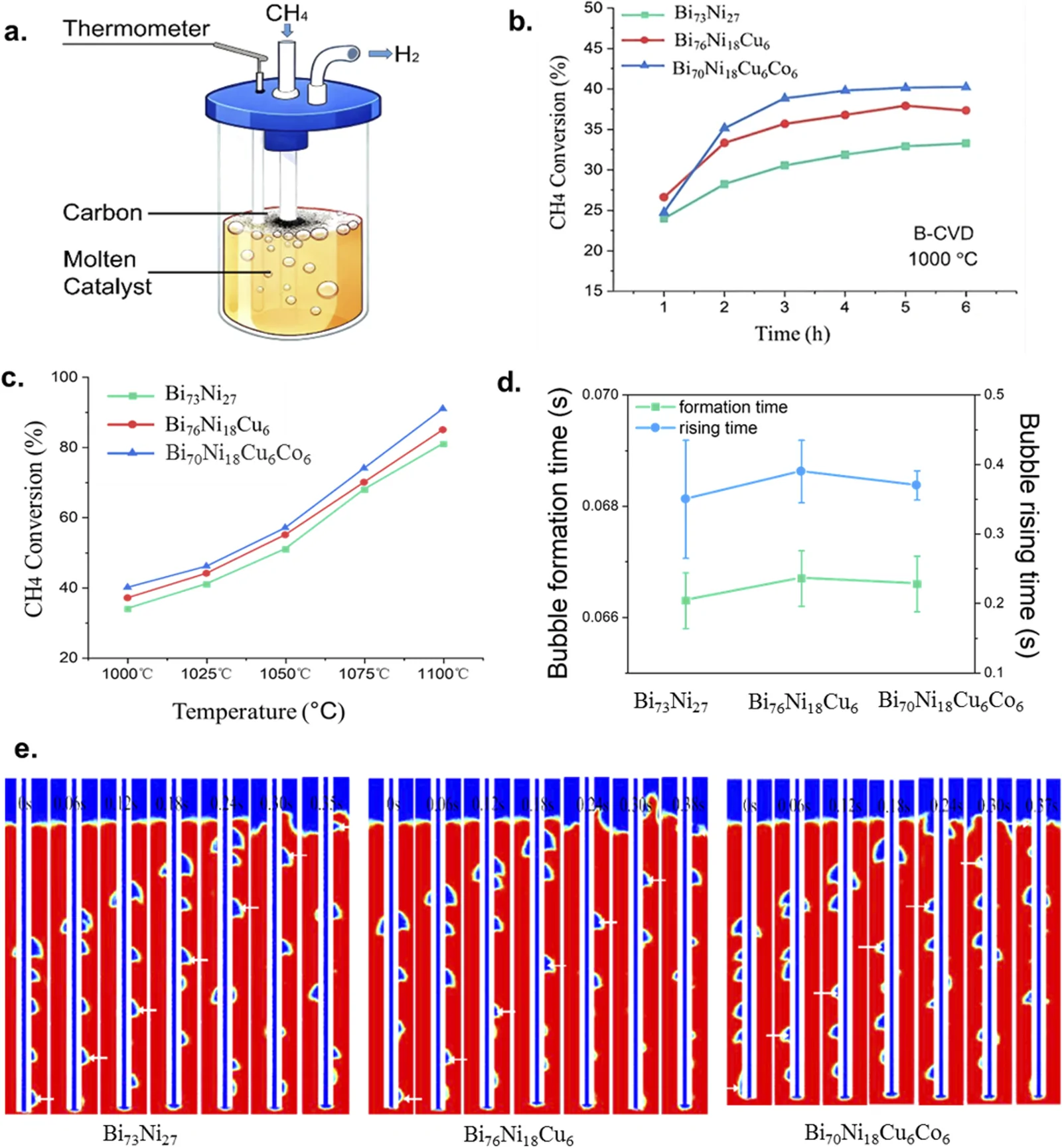
Experimental test for CH_4_ pyrolysis and bubble dynamic simulation. (a) Illustration of a molten bubble CVD experimental setup; (b) the CH_4_ conversion efficiencies of Bi_73_Ni_27_, Bi_76_Ni_18_Cu_6_, and Bi_70_Ni_18_Cu_6_Co_6_ over a 6 h reaction period; (c) comparison of the CH_4_ conversion efficiencies of Bi_73_Ni_27_, Bi_76_Ni_18_Cu_6_, and Bi_70_Ni_18_Cu_6_Co_6_ at different temperatures of 1000–1100 °C; (d) the CFD-simulated bubble formation and rising times in the Bi_73_Ni_27_, Bi_76_Ni_18_Cu_6_, and Bi_70_Ni_18_Cu_6_Co_6_ molten alloys. (e) Simulated bubble rise behavior by computational fluid dynamics in different molten catalysts: Bi_73_Ni_27_, Bi_76_Ni_18_Cu_6_, and Bi_70_Ni_18_Cu_6_Co_6_. The arrows highlight the rising and coalescence processes of a single bubble.

Based on the Bi_70_Ni_18_Cu_6_Co_6_ model ratios, we conducted experimental tests and compared the results with those of Bi_73_Ni_27_ and Bi_76_Ni_18_Cu_6_ under the same conditions. As shown in Fig. 3b, the initial increase in CH_4_ conversion during the first 1–3 h can be attributed to an induction period associated with gradual surface reconstruction and equilibration of the molten alloy. During this stage, continuous gas bubbling promotes dynamic redistribution of solute atoms, leading to progressive exposure of catalytically active sites. When it arrives at equilibrium (3–6 h), the results of Bi_70_Ni_18_Cu_6_Co_6_ can achieve 40.3% CH_4_ conversion at a moderate 1000 °C, outperforming Bi_73_Ni_27_ (33.3%) and Bi_76_Ni_18_Cu_6_ (37.4%) molten media under the same experimental conditions.^14,17^ (Notably, here the obtained values deviate from previous reports due to variations in experimental conditions such as reactor insulation, CH_4_ concentration, and CH_4_ residence time in the molten medium). Compared to Bi_73_Ni_27_, the added Cu/Co components favored the enhancement of catalytic performance for CH_4_ cracking. To validate this change, we further estimated their CH_4_ conversion efficiencies at the chosen values of 1000 °C, 1025 °C, 1050 °C, 1075 °C, and 1100 °C (Fig. 3c). A similar increasing trend is observed; however, the differences among them become progressively smaller. This is attributed to the endothermic nature of CH_4_ cracking, where thermal cracking becomes the dominant pathway near 1100 °C. In addition, we fitted Arrhenius plots with 1/*RT* as the *x*-axis to estimate the apparent activation energy at 1000 °C (Fig. S7). The obtained activation energy value (197.3 kJ mol^−1^) of Bi_70_Ni_18_Cu_6_Co_6_ is lower than that of Bi_73_Ni_27_ (224.2 kJ mol^−1^) and Bi_76_Ni_18_Cu_6_ (208.8 kJ mol^−1^). Compared with these catalysts, these data validate that upon the introduction of the screened promoter metal Co, the obtained Bi_70_Ni_18_Cu_6_Co_6_ exhibits superior activity for CH_4_ pyrolysis.

Besides the intrinsic catalytic activity of the molten metal, bubble dynamics (formation and rising behavior) in the B-CVD method, which determine the reaction environment and time, also play a critical role in CH_4_ pyrolysis in melt. Thus, we performed computational fluid dynamics (CFD) to simulate the gas–liquid two phase flow inside the reactor and quantify bubble volume, formation/detachment period, residence time, and coalescence statistics. As shown in Fig. 3d, the obtained quantitative data show that the average bubble formation times and rising times of these catalysts are very close to each other. As shown in Fig. 3e, the CFD-simulated bubble rise behaviors in the Bi_73_Ni_27_, Bi_76_Ni_18_Cu_6_, and Bi_70_Ni_18_Cu_6_Co_6_ catalysts were very similar, with no significant difference observed (for more details, see the CFD simulation in SI, Fig. S8 and Table S3). These results exclude the potential influence of bubble dynamics on the difference in CH_4_ conversion among these catalysts and confirm the improved intrinsic catalytic activity of quaternary Bi_70_Ni_18_Cu_6_Co_6_.

### 3.4 Product characterization

Following the CH_4_ pyrolysis reaction by Bi_70_Ni_18_Cu_6_Co_6_, gas-phase products were quantitatively analyzed by gas chromatography, confirming H_2_ as the dominant gaseous product with small amounts of C_2_ and C_3_ hydrocarbons, while the solid carbon powder product was separated from the molten catalyst, collected, and characterized. Fig. 4a illustrates the XRD pattern of the generated carbon powder. Two intense peaks centered at 2*θ* ∼ 26.5° are identified, which are typical features of graphene layers, while a weak peak centered at 2*θ* ∼ 24.6° is attributed to graphite. In addition, the structural features of the carbon powders were characterized by Raman spectra (Fig. 4b). Qualitative analysis was performed using the intensity ratio of the D band (∼1350 cm^−1^) to the G band (∼1580 cm^−1^), *I*_D_/*I*_G_, which ranged from 0.68 to 0.73 across different carbon samples. Raman spectroscopy indicated that most of the carbon powder was graphene [*I*_D_/*I*_G_ > 0.7], which can be compared to the majority of commercial graphene in a range of 0.7–2. The number of layers present in graphene can also be determined by using the full width at half-maximum (82.77–86.25 cm^−1^) of the 2D peak, which is approximately 12–16 sheets. In summary, the solid carbon yield was consistent with methane conversion within experimental error, confirming an overall carbon–hydrogen balance close to unity.

**Fig. 4:**
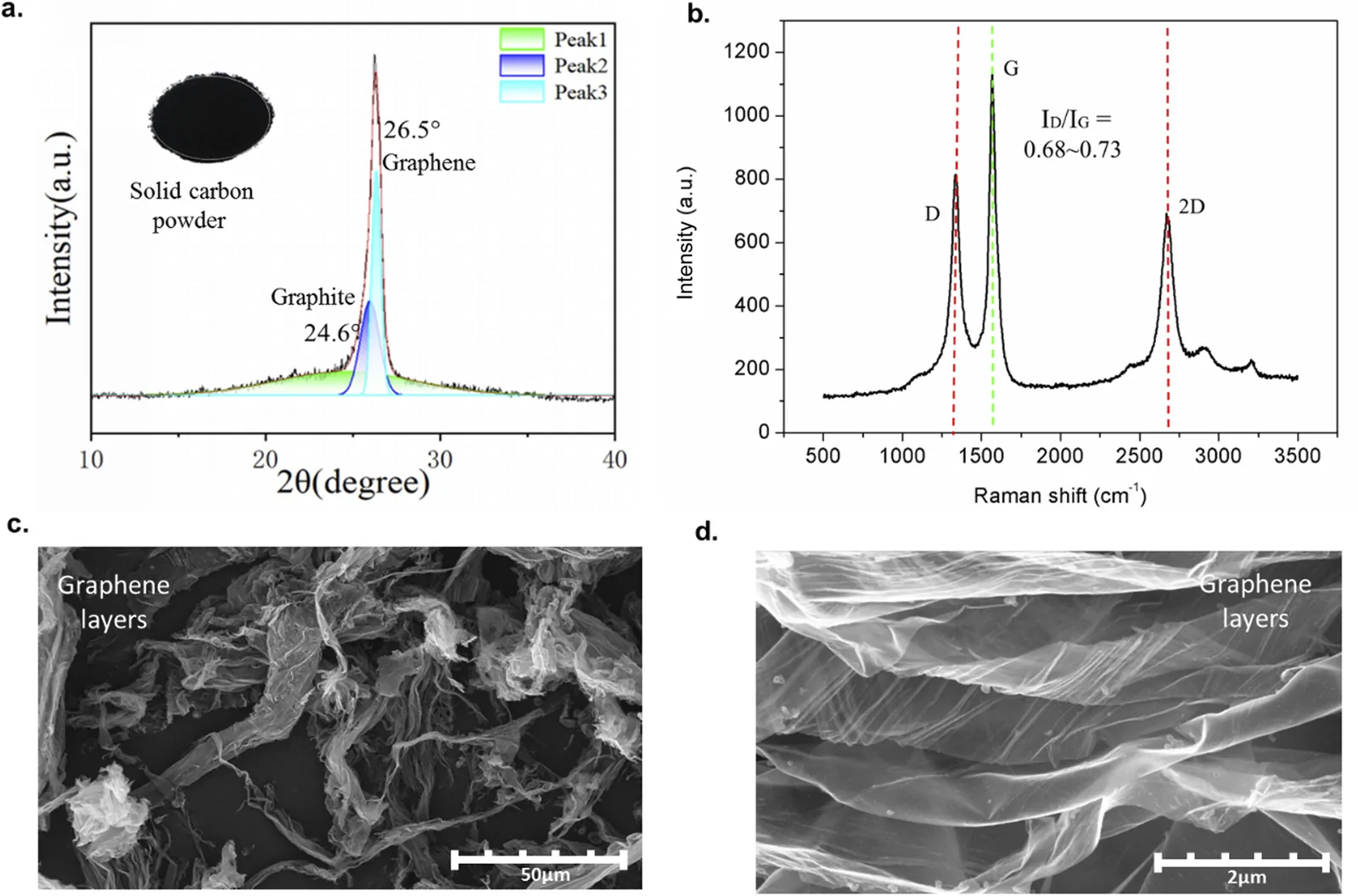
Carbon characterization of the reaction products. (a) XRD profiles of carbon powder, showing three sub-peaks, with peak 1 corresponding to graphite, and peak 2 and peak 3 corresponding to graphene layers with different stacking. (b) Raman spectra of carbon powder. (c) and (d) The SEM image of graphene at the scales of 50 µm (c) and 2 µm (d), respectively.

Moreover, we adopted scanning electron microscopy (SEM) to characterize the graphene. As shown in Fig. 4c and d, the low-/high-magnification SEM images reveal that the graphene synthesized *via* this process exhibits a typical lamellar stacking structure with distinct and well-defined layer edges. At low magnification (scale ∼ 50 µm, Fig. 4c), the sample surface displays pronounced layered and wrinkled features, accompanied by partial layer stacking and uneven deposition, indicating macroscopic undulations within the graphene sheets. High-magnification observations (scale ∼ 2 µm, Fig. 4d) show a smoother surface morphology with clearly defined lamellar edges and no visible particle residues, suggesting high surface cleanliness and excellent film continuity. Overall, this graphene exhibits high lamellar integrity and structural continuity, fulfilling the quality requirements for potential applications such as conductive films or catalytic supports and demonstrating promise as a high-value carbon material.^46,47^

### 3.5 Microscopic catalytic kinetics simulation

To characterize the microkinetic activity of Bi_70_Ni_18_Cu_6_Co_6_ in CH_4_ pyrolysis at the atomic scale, AIMD/DFT-based simulations were conducted. In the AIMD simulations, snapshots of the Bi_70_Ni_18_Cu_6_Co_6_ alloy were extracted at different steps of AIMD trajectories. From these, candidate surfaces with sufficient exposure of solute metal Ni/Cu/Co atoms were selected for slab model construction and subsequent geometry optimization (Fig. S9). Post-optimization, the adsorption energies of CH_3_ groups and single H atoms at various metallic sites were calculated. For CH_3_ adsorption, the lowest adsorption energy was observed on Bi sites, with the highest value of −2.387 eV, indicating a relatively strong binding affinity of Bi toward methyl species. For H adsorption, solute metal sites such as Ni, Cu, and Co generally exhibit lower adsorption energies than Bi sites, with the highest value of −2.289 eV for the Co site, suggesting that H atoms preferentially bind to these solute metals. When H atoms were modeled to penetrate into the bulk molten phase, the value of adsorption energy decreased, indicating decreased stability and a clear tendency for hydrogen to remain on the surface rather than entering the bulk.^48^

CH_4_ pyrolysis proceeds through a sequential dehydrogenation pathway involving CH_4_ → CH_3_ + H, followed by further hydrogen removal steps (CH_3_ → CH_2_ → CH → C). In this work, we focus on the initial C–H bond activation step because it represents the key initiation process of methane dissociation. Due to the high thermodynamic stability of CH_4_, cleavage of the first C–H bond generally requires substantial energy input and strongly determines the intrinsic methane activation capability of catalysts. In the surface configuration of Bi_70_Ni_18_Cu_6_Co_6_, four representative C–H bond dissociation pathways were considered and computed for activation energy comparison (Fig. 5a and b): (A) hydrogen adsorbed on Ni and CH_3_ adsorbed on Bi; (B) hydrogen and CH_3_ both adsorbed on Bi; (C) hydrogen adsorbed on Cu and CH_3_ adsorbed on Bi; (D) hydrogen adsorbed on Co and CH_3_ adsorbed on Bi. The results revealed that pathway D (CH_4_ → CH_3_–Bi + H–Co) had the lowest reaction activation energy. Upon incorporation of Co, the minimum C–H dissociation barrier decreased to 1.96 eV, markedly lower than that in the absence of Co. Transition-state geometry analysis indicated that Co possesses stronger H* adsorption capability and promotes hydrogen migration between active sites, thus accelerating the initial CH_4_ dissociation step.

**Fig. 5:**
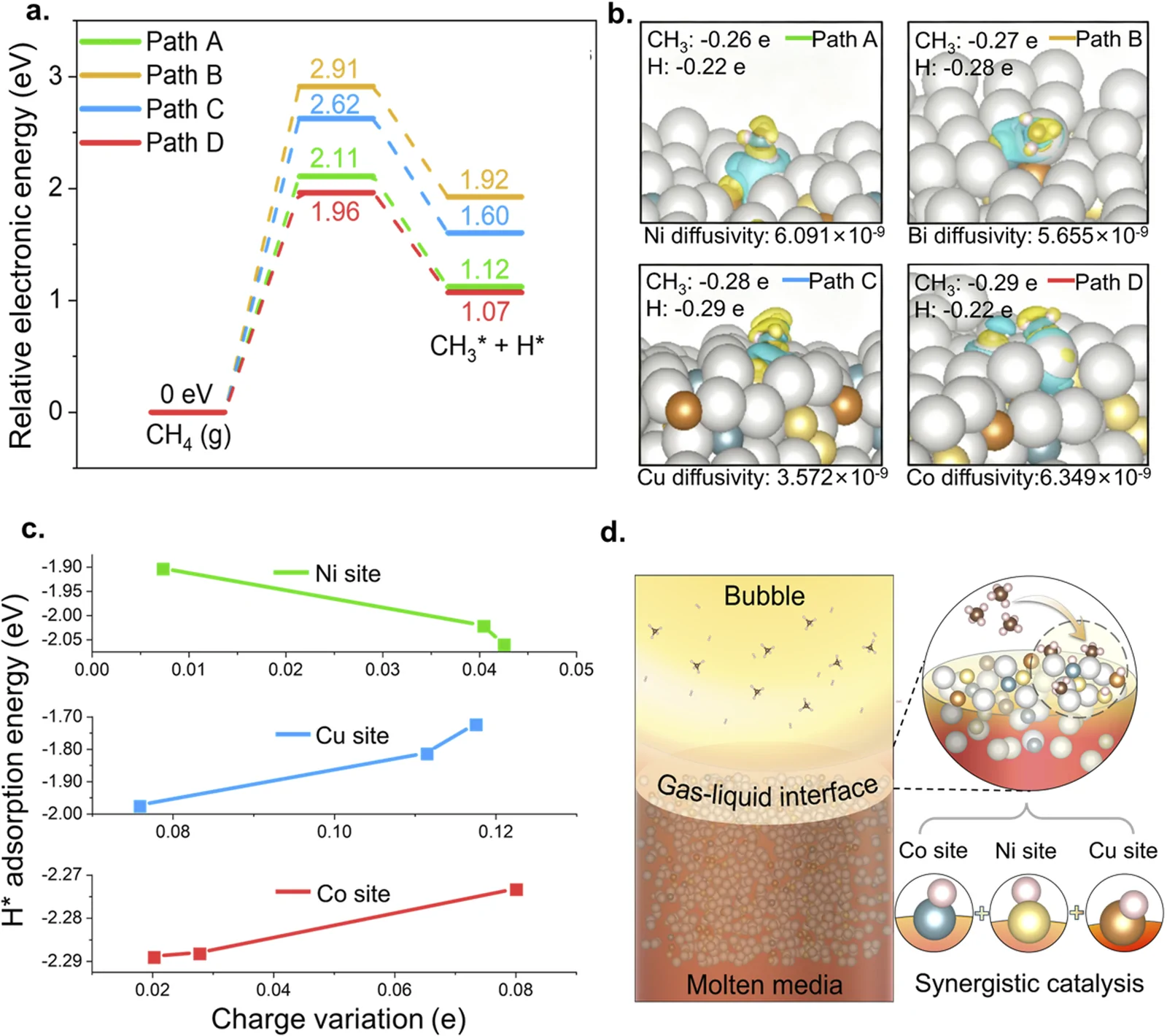
Surface state simulations and activity analysis of Bi_70_Ni_18_Cu_6_Co_6_ molten catalysts for CH_4_ pyrolysis. (a) Activation energy diagram of C–H bond dissociation pathways on the Bi_70_Ni_18_Cu_6_Co_6_ alloy surface. (b) Charge-transfer amounts of 
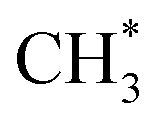
 and H* along different adsorption pathways and the diffusion coefficients of the corresponding adsorbed atoms obtained from AIMD simulations. (c) Comparison of the Bader charge differences of surface metal atoms in various quaternary Bi_70_Ni_18_Cu_6_Co_6_ alloy systems before and after H adsorption, and their correlation with the corresponding adsorption energies. (d) Schematic illustration of a synergistic catalytic effect in the multi-solute-metal Bi_70_Ni_18_Cu_6_Co_6_ molten catalyst.

To evaluate how molten alloy diversification influences diffusion and surface exposure, the diffusion coefficients of individual metal atoms and the average diffusion coefficient were calculated for the Bi_73_Ni_27_, Bi_76_Ni_18_Cu_6_, and Bi_70_Ni_18_Cu_6_Co_6_ molten alloys (Table S4). The results show that the incorporated Co exhibits a high diffusion coefficient (6.349 × 10^−9^ m^2^ s^−1^), comparable to that of Ni, and simultaneously increases the overall diffusion coefficient from 5.108 × 10^−9^ m^2^ s^−1^ in Bi_76_Ni_18_Cu_6_ to 5.652 × 10^−9^ m^2^ s^−1^ in Bi_70_Ni_18_Cu_6_Co_6_. This enhanced atomic mobility leads to a more rapid and uniform redistribution of solute metals between the bulk and surface. Such dynamic benefits enlarge the probability of active-site exposure to CH_4_ bubbles during the reaction, thereby directly contributing to the improved catalytic performance of the quaternary system.

In order to elucidate the relationship between adsorption energy and charge transfer, metal exposed surface models with different H adsorption sites were evaluated. The charge density differences associated with adsorbed H atoms and CH_3_ species were examined, revealing minimal variation in charge transfer among the optimized stable adsorption configurations. We subsequently analyzed the net Bader charge differences of the primary adsorption-site metal atoms before and after adsorption (Table S5). A strong correlation was observed between the magnitude of adsorption energy and the extent of charge transfer (Fig. 5c). For all metal atoms, H adsorption decreased the net charge on the corresponding metal sites, indicating electron transfer from the metal to H. This behavior brings the metal atoms closer to charge neutrality and causes the hydrogen species to acquire partial negative charge, resembling σ-bond polarization commonly observed in metal hydride formation. This phenomenon aligns with previously reported correlations between Bader charge variation and catalytic performance. For the active metal Ni, larger net charge differences correspond to higher adsorption energies, consistent with strong metal–H interaction.^49^ Although Co and Cu retain catalytic activity toward CH_4_ cracking similar to Ni, the promoter metals Co and Cu exhibit an opposite trend, in which greater charge transfer is associated with lower adsorption energies. The increased charge redistribution lowers hydrogen adsorption strength, facilitating hydrogen detachment and subsequent H_2_ formation. These results clarify the role of promoter metals in assisting active metals by mitigating excessive adsorption strength and thus enabling more favorable hydrogen release and dehydrogenation pathways (Fig. 5d).

### 3.6 Active mechanism analysis

Recent studies on methane and light-alkane oxidation have shown that electronically coupled active sites, current-induced charge redistribution, and engineered metal–support interfaces can substantially alter C–H activation and subsequent reaction steps. Although these systems involve oxidative catalysis rather than methane pyrolysis, they further illustrate the general importance of cooperative electronic interactions in catalyst design.^50–52^ Based on the binary Bi–Ni molten systems, the catalyst properties of the molten system can be modified with additional Cu and/or Co elements, similar to how soluble metal complex catalysts can be modified by tuning ligands to modify active sites and their interaction with solvents. Through the introduction of Co, the proposed Bi_70_Ni_18_Cu_6_Co_6_ catalyst can successfully increase the catalysis activity of the molten system for CH_4_ pyrolysis, as a result of Co adjusting the interactions between the active metal and solute metals. Based on the initial Bi_70_Ni_18_Cu_6_Co_6_ model ratios, we conducted experimental tests by changing the ratio of Co (*X* = 1–6%, Bi_70+*X*_Ni_18_Cu_6_Co_6−*X*_) to investigate the influence of Co concentration on catalytic performance, as shown in Fig. S10. The results demonstrate that the catalytic activity varies with Co concentration, and the optimized Bi_73_Ni_18_Cu_6_Co_3_ composition achieves a higher CH_4_ conversion efficiency than the initial Bi_70_Ni_18_Cu_6_Co_6_ catalyst. Nevertheless, the overall variation among different Co concentrations is relatively limited, indicating that the underlying micro-mechanism could be attributed to a synergistic catalytic effect rather than Co concentration alone. In this multi-solute molten system, solute metal Ni, Cu, and Co atoms exhibit pronounced inter-solubility and high diffusion in a Bi solvent with low surface tension. This multi-solute combination enhances the exposed active sites and offers more opportunities for direct interactions with CH_4_ molecules. Meanwhile, the high diffusion multi-solute system can push the solute out of a “cage effect” between Bi atoms and surrounding atoms, avoiding this obstacle to CH_4_ molecule dissociation.

Additionally, the additional charge induced by Co can change the charge distribution in the molten system, which is positively correlated with the catalytic activity of molten systems for CH_4_ pyrolysis. A possible explanation for this correlation is that electrons from solute metals move rapidly to fill antibonding orbitals in the CH_4_ molecule, which weakens the C–H bonds, reducing the activation energy as confirmed by the apparent activation energy in experiments and DFT simulations. To quantify the bonding strength changes of the C–H bonds, the integrated crystal orbital Hamiltonian population (ICOHP) was calculated and compared for a free CH_4_ molecule and a Co-added CH_4_ molecule model, respectively. In the molten catalyst, CH_4_ pyrolysis happens at a bubble–liquid interface. It is necessary to consider the solvent effect. To address this, the implicit solvation model is employed to correct the Co adsorption free energies. The free CH_4_ possesses a tetrahedral molecular structure, consisting of four equivalent C–H bonds with a bond length of 1.09 Å. Under the influence of Co, its structure undergoes slight distortion and the bond length is elongated to 1.10 Å. The C–H bond strength is directly proportional to the magnitude of the negative ICOHP, with larger negative values signifying stronger bonding interactions. By comparison, as shown in Fig. 6a and b, the ICOHP value becomes less negative after Co incorporation, changing from −5.13 eV to −5.03 eV, indicating an overall weakening of the C–H bond interaction. This weakened bonding strength facilitates C–H bond cleavage during methane activation. Furthermore, Co incorporation introduces more accessible C–H antibonding states above the Fermi level, which facilitates electron transfer into antibonding orbitals and provides an electronic origin for the weakened C–H interaction. The increased antibonding-state availability contributes to the destabilization of C–H bonds, consistent with the weakened C–H interaction revealed by ICOHP analysis.

**Fig. 6:**
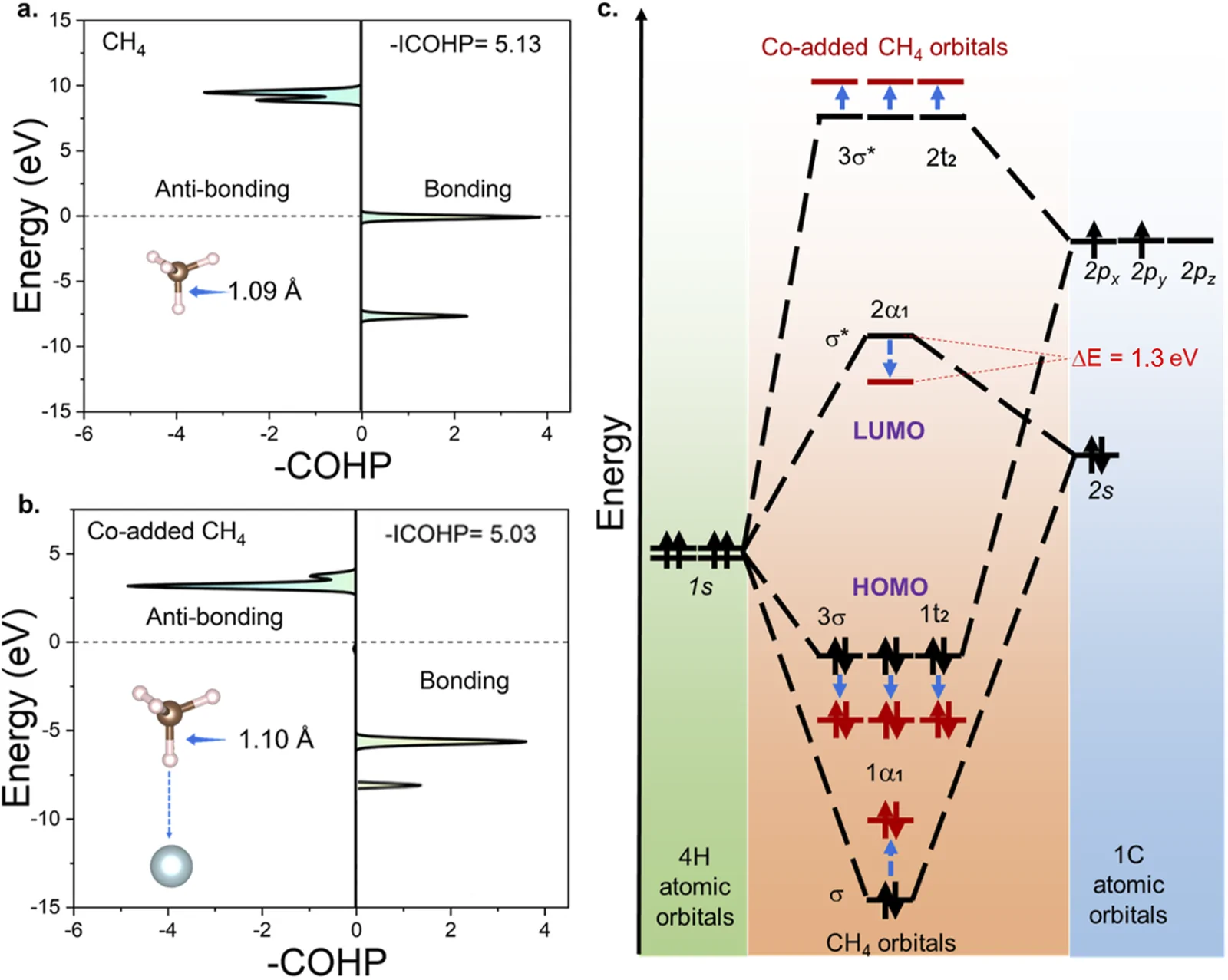
The bonding strength and orbital energy analysis for a CH_4_ molecule under the effect of Co. (a) and (b) The –ICOHP bonding characteristics as a function of energy for a free CH_4_ molecule (a) and a CH_4_ molecule surrounded by one Co atom (b), respectively. (c) The energy level diagram for a CH_4_ molecule and Co-added CH_4_ molecule. The black and blue lines represent the atomic energy level of a CH_4_ molecule and Co-added CH_4_ molecule, respectively.

The Co activation for CH_4_ pyrolysis can also be revealed by the energy diagram that features the C–H bonding characters (see Fig. 6c). The free CH_4_ has triply degenerate molecular orbitals produced as a result of the overlap of two 2p carbon orbitals and three 1s orbitals of H atoms, and all electrons occupy bonding molecular orbitals with an electronic configuration of (1a_1_)^2^(1t_2_)^6^(2a_1_)^0^. Under normal conditions, the absence of low-lying empty orbitals (σ molecular orbitals) and high-energy filled orbitals (σ* molecular orbitals) makes the participation of free CH_4_ in the pyrolysis reaction tough. In comparison, the energy of the lowest unoccupied σ* molecular orbital (LUMO) of CH_4_ in the Co-incorporated solvation model decreases by ∼1.3 eV (notably, this value may deviate from that under realistic molten-state conditions), making electron occupation of the CH_4_ LUMO relatively easier. Additionally, a highly negative electron affinity (−1.9 eV) indicates that the CH^4−^ anion is less stable than CH_4_ itself. As shown in Fig. 6b, the charge transfer between 
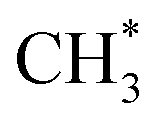
 and the Co-based catalyst surface state creates conditions for filling antibonding HOMO orbitals. Collectively, the Co-induced downshift of the LUMO of CH_4_, coupled with charge antibonding occupation, weakens C–H bonds, thereby accelerating the overall CH_4_ pyrolysis process.

### 3.7 Engineering feasibility and data feedback toward closed-loop catalyst discovery

The practical feasibility of the designed Bi–Ni–Cu–Co molten catalyst was further evaluated. Although the quaternary alloy exhibits enhanced catalytic activity for CH_4_ pyrolysis, several engineering challenges remain for practical applications. The incorporation of Co improves CH_4_ activation through optimized charge transfer, hydrogen adsorption, and atomic diffusion; however, its relatively high melting point increases the difficulty of catalyst preparation and homogenization under practical conditions. Moreover, the multicomponent molten system introduces additional challenges in the separation of solid carbon products from the liquid alloy phase. In the current study, carbon products were collected by scraping the carbon materials floating on the surface of the molten alloy catalyst after CH_4_ pyrolysis reactions, rather than being continuously separated from the molten alloy. Therefore, the proposed Bi–Ni–Cu–Co system should be considered as a high-performance model catalyst demonstrating the effectiveness of descriptor-guided design, while further optimization of promoter elements is required to achieve practical deployment.

Importantly, the experimental and theoretical results obtained from this catalyst system provide valuable feedback for continuous optimization of the DigMethpy platform. The measured catalytic performance, alloy composition, reaction conditions, carbon products, and kinetic parameters can be integrated into the experimental database to enrich the knowledge base for future catalyst exploration. Meanwhile, AIMD/DFT-derived descriptors, including solute diffusion coefficients, Bader charge transfer, H* adsorption energies, and C–H bond activation characteristics, provide theoretical guidance for refining descriptor–activity relationships. Through this iterative feedback process, newly acquired experimental and computational data can update descriptor criteria and improve predictive accuracy, enabling DigMethpy to evolve from a static screening platform into an adaptive closed-loop framework for rational discovery of scalable molten catalysts.

## 4. Conclusions

In summary, this study presents a descriptor-based design strategy comprising front-end data-driven exploration of potential catalysts, mid-end experimental synthesis and testing, and back-end theoretical mechanistic analysis to identify promising quaternary molten alloy catalysts for CH_4_ pyrolysis. Adopting three physically motivated activity-descriptors: solute-metal diffusion coefficient, Bader charge transfer, and H* adsorption energy, we simulated descriptor data of Bi_70_Ni_18_Cu_6_X_6_ in different solute metal X systems and identified Co as the optimal constituent metal. In the experiment, we performed component optimization and obtained a superior Bi_70_Ni_18_Cu_6_Co_6_ catalyst with a high CH_4_ conversion efficiency of ∼40.3%. The underlying mechanism of the multicomponent molten metal was elucidated by the synergistic catalytic effect of multiple solutes. Co modifies the local charge environment, promoting electron transfer from the catalyst to the antibonding orbitals of CH_4_. According to ICOHP and molecular orbital energy level analysis, Co induces a substantial downshift (∼1.3 eV) of the LUMO energy level of CH_4_, which facilitates electron addition to the antibonding LUMO state of CH_4_, making C–H bond cleavage energetically more accessible, thereby improving the CH_4_ dissociation efficiency. This work not only provides a promising catalyst system for CH_4_ pyrolysis, contributing to the advancement of the hydrogen society, but also promotes a descriptor-empowered multicomponent molten catalyst design for CH_4_ pyrolysis.

## Author contributions

Hangwei Liu and Zihao Cheng performed the research and wrote the paper. Xuxuan Huang, Dong Zhao, Junmei Du, and Teli Lin analyzed the data. Jinwen Li and Zhaoyu Huang performed the CFD simulation. Yangdong He, Wenhan Wang, Wei Yang, and Seok Ki Kim offered formal analysis. Hao Li and Yongliang Tang provide resources and revised the manuscript. Yuanzheng Chen designed the study and revised the manuscript.

## Conflicts of interest

The authors declare no competing interests.

## Supplementary Material

SC-017-D6SC05383A-s001

## Data Availability

The data that support the findings of this study are available from the corresponding author upon reasonable request. Supplementary information (SI): details of descriptor identification through the DigMethpy module, simulation-based calculation of descriptor data, the CFD simulation of bubble behavior, and microscopic catalytic simulation. See DOI: https://doi.org/10.1039/d6sc05383a.
