# Supplementary material for: A closed-loop framework for descriptor-empowered exploration of high-activity Co-based quaternary molten alloy catalysts for methane pyrolysis
Source: Chem Sci. 2026 Sep 3;17(38):18139–51. doi: 10.1039/d6sc05383a (PMC13551427; doi:10.1039/d6sc05383a)
Supplement: Supplementary file 1 [file SC-017-D6SC05383A-s001.pdf]

Supplementary Information (SI) for Chemical Science.
This journal is © The Royal Society of Chemistry 2026

Supporting Information

Closed-Loop Framework for Descriptor-Empowered Exploring High-Activity Co-Based Quaternary Molten Alloys Catalysts for Methane Pyrolysis

Hangwei Liu[1], Zihao Cheng[1], Xuxuan Huang[1], Yangdong He[2], Wenhan Wang[2], Wei Yang[2], Jinwen Li[1], Zhaoyu Huang[1], Dong Zhao[1], Junmei Du[1], Teli Lin[1], Seok Ki Kim[4], Hao Li[3,*], Yongliang Tang[1,*], Yuanzheng Chen[1,*]

[1]*School of Physical Science and Technology, Southwest Jiaotong University, Chengdu, Sichuan 610031, China.*

[2]*Research Institute of Natural Gas Technology, PetroChina Southwest Oil & Gasfield Company, Chengdu, 610213, P.R. China.*

[3]*Advanced Institute for Materials Research (WPI-AIMR), Tohoku University, Sendai, 980-8577, Japan.*

[4]*Department of Energy Systems Research, Ajou University, Suwon 16499, Republic of Korea.*

*Email: li.hao.b8@tohoku.ac.jp; tyl@swjtu.edu.cn; cyz@swjtu.edu.cn

Contents:

1. Descriptor identification through the DigMethpy module 1

2. Simulation-based calculation of descriptor data....................................3

3. Experimental setup and validation..8

4. The CFD simulation of bubble behavior 9

5. Microscopic catalytic simulation 11

1. Descriptor identification through the DigMethpy module

To identify the intrinsic factors governing the catalytic activity of molten metal systems for methane pyrolysis, the “Descriptors” module implemented in DigMethpy was developed to perform data-driven feature engineering and descriptor extraction. A curated dataset containing reported catalytic performances of molten metal catalysts for methane pyrolysis was first established by integrating experimental data from literature and theoretical calculations. The dataset includes catalyst compositions, reaction conditions, methane conversion efficiency, and relevant physicochemical properties of constituent metal elements.

More than 20 candidate physicochemical features were collected and evaluated, including electronic properties (e.g., Bader charge, electronegativity, ionization energy, orbital electron number), atomic properties (e.g., atomic radius, atomic mass, density), molten-state dynamic properties (e.g., solute diffusion coefficient, viscosity, surface tension), and catalytic interaction parameters (e.g., H* adsorption energy and interface energy). These physicochemical features were systematically analyzed through correlation analysis, feature importance evaluation, and machine-learning-assisted feature selection to identify the most representative parameters associated with methane pyrolysis activity.

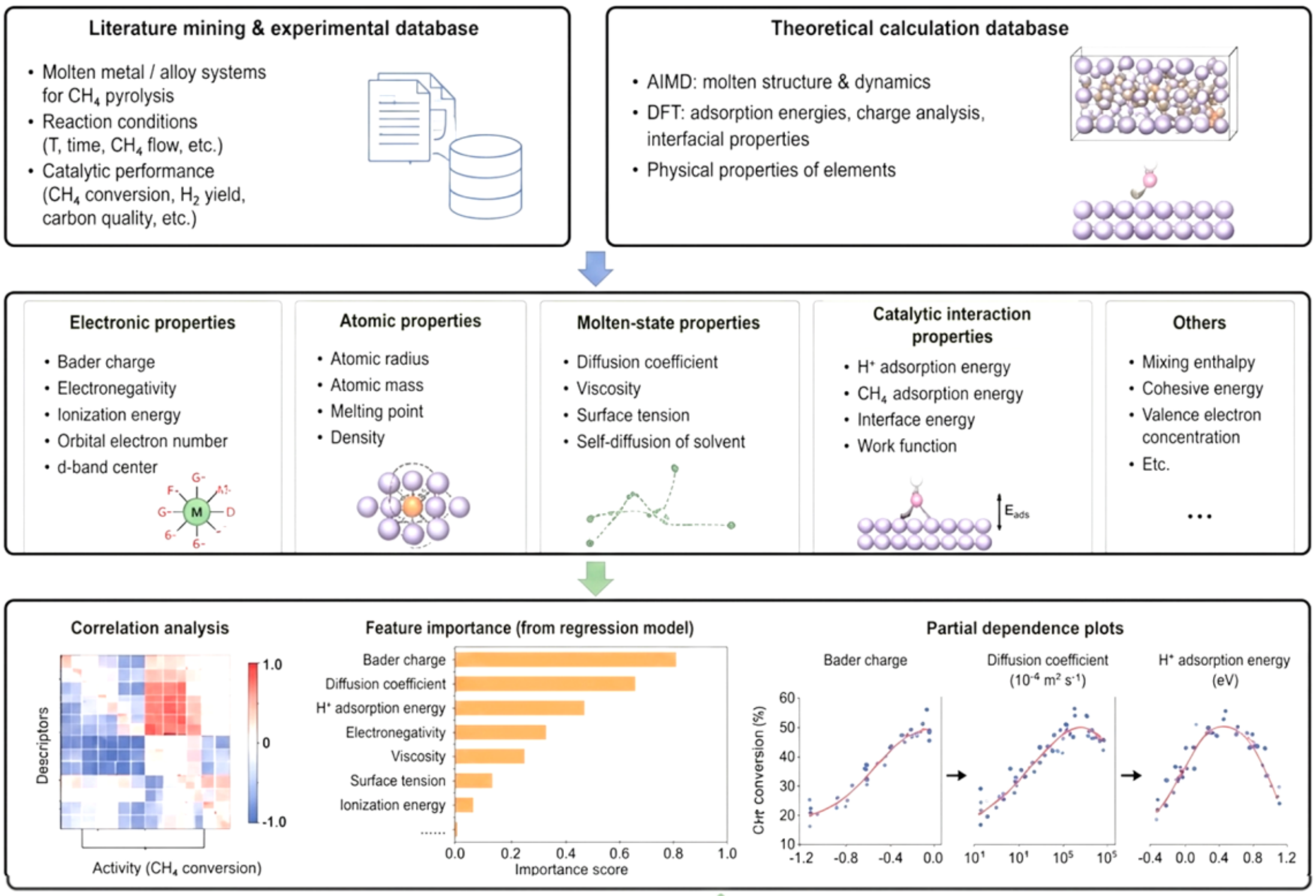

Figure S1. Descriptor discovery workflow implemented in DigMethpy for molten methane pyrolysis catalysts. Experimental and theoretical databases were integrated to construct a multidimensional descriptor pool containing electronic, atomic, molten-state, and catalytic interaction properties. The data analysis involved correlation analysis, feature importance evaluation, and partial dependence analysis.

The analysis revealed that three key descriptors exhibited strong physical correlations with catalytic performance in molten metal systems: (i) the Bader charge of active solute metals, (ii) the diffusion coefficient of solute atoms in the molten phase, and (iii) the H adsorption energy on solute metal surfaces. The Bader charge descriptor reflects the electronic redistribution between active metal components and reaction intermediates, which directly influences C–H bond activation. The solute diffusion coefficient describes the dynamic mobility of catalytic species in the molten environment, representing the accessibility and regeneration capability of active sites. The H* adsorption energy characterizes the balance between hydrogen adsorption and desorption, which is critical for efficient hydrogen evolution and catalyst surface regeneration.

To validate the effectiveness and physical relevance of the descriptors identified by the DigMethpy descriptor module. Using binary Bi-based alloy systems as representative cases, the module was applied to evaluate the relationships between CH_4 pyrolysis activity and the three proposed descriptors, as shown Figure S2. As shown in the left panel, the CH_4 conversion exhibits a strong positive correlation with the Bader charge of the solute metal ($R^2 = 0.90$). The observed correlation trend is in good agreement with the experimentally reported relationships in previous studies [*Science* 358, 917 (2017)]. Meanwhile, A strong correlation (R^2) is also observed between catalytic activity and atomic mobility of solute species and H* adsorption energy. Unlike the linear correlations observed for Bader charge and diffusion coefficient, H* adsorption energy exhibits a volcano-type relationship ($R^2 = 0.77$), suggesting an optimal hydrogen binding strength for methane pyrolysis catalysis.

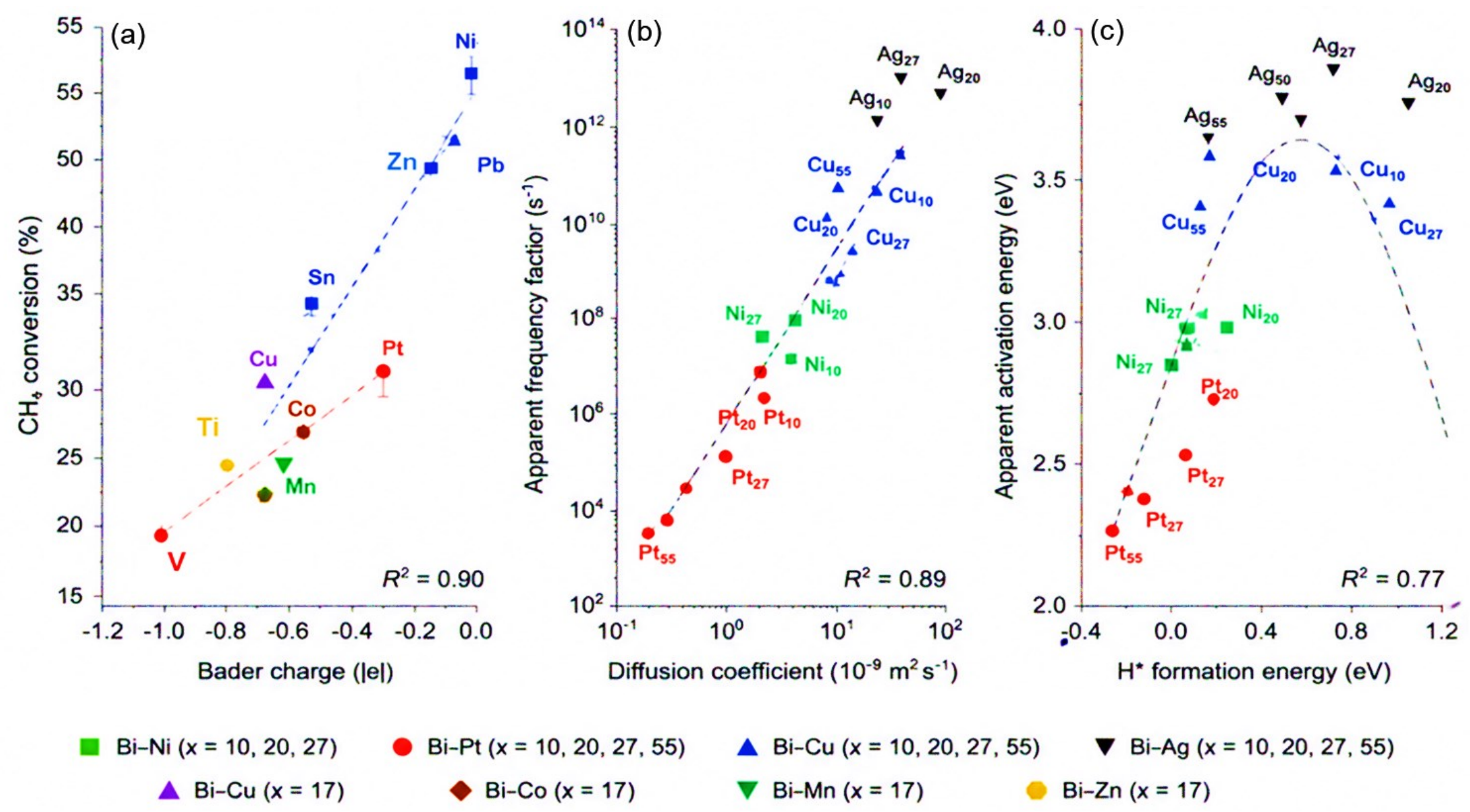

Figure S2. Validation of DigMethpy-derived catalytic descriptors using binary Bi-based molten alloy (Bi-M) systems. The coordinates were labeled as Mx, where M denotes the solute metal and X in Mx denotes the atomic concentration of the solute metal in Bi solvent. (a) Correlation between CH_4 conversion and solute Bader charge of different Bi-based alloys. (b) Relationship between CH_4 conversion and solute diffusion coefficient in molten Bi alloys. (c) Volcano-type correlation between CH_4 conversion and H adsorption energy.

Overall, the data correlation of Bi-M systems in Figure S2 confirms that the catalytic activity of molten Bi-based alloys positively correlate with three fundamental factors. These results validate the effectiveness of the DigMethpy descriptor module in extracting physically meaningful catalytic descriptors and provide a rational basis for descriptor-guided discovery and optimization of molten methane pyrolysis catalysts. It also should be noted that some of these values were obtained through machine-learning-assisted prediction rather than direct experimental measurements, and therefore may involve certain prediction errors.

2. Simulation-based calculation of descriptor data

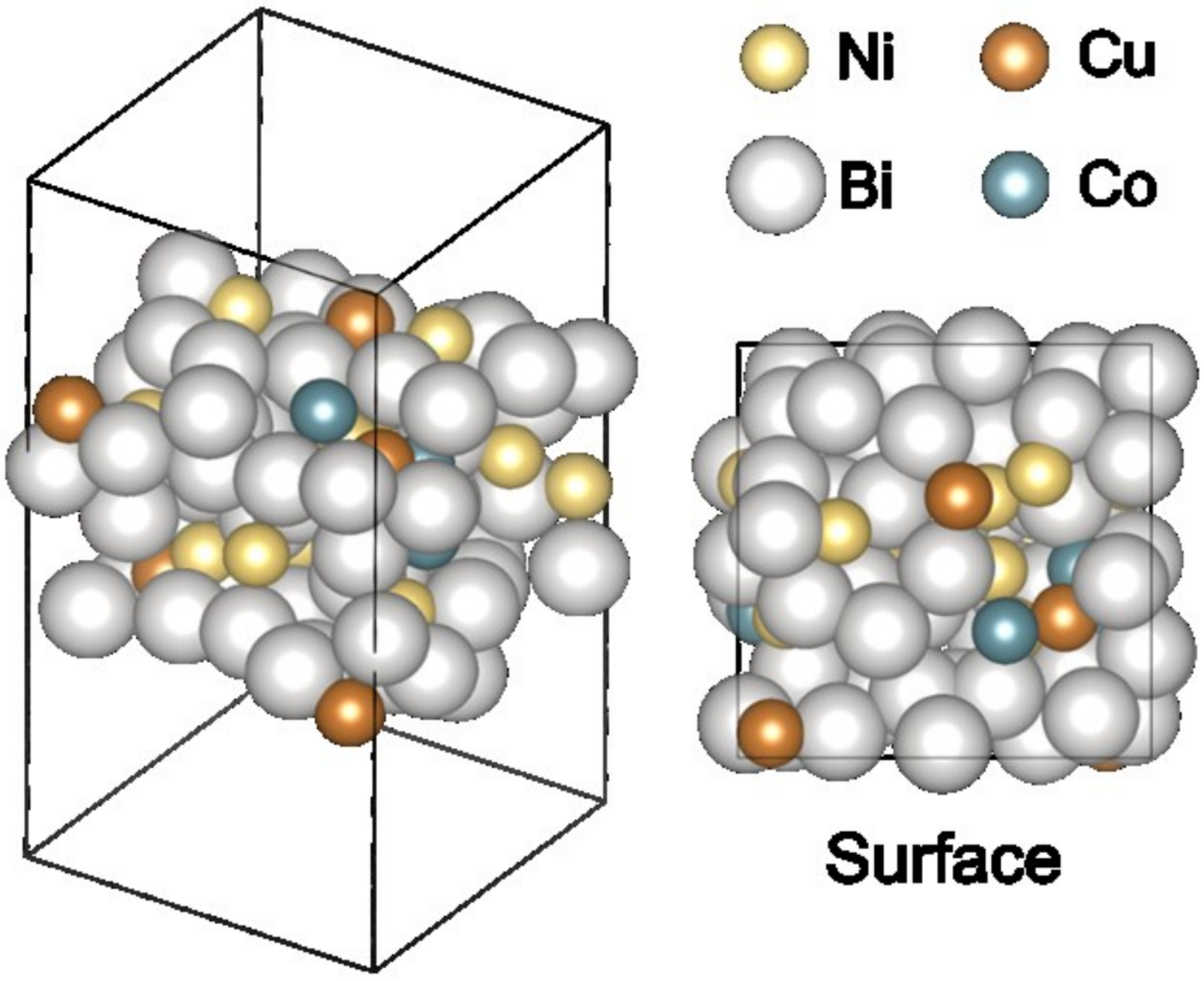

Figure S3. The $Bi_{70}Ni_{18}Cu_6X_6$ alloy model was constructed and used as the initial configuration. Structural optimization was performed prior to the AIMD simulations to ensure the model to relax into an energetically optimized configuration. Due to the high computational cost of first-principles calculations, the present AIMD simulations are limited by the available simulation timescale (1500 K, 10 ps) and model size (< 200 atoms), thus the obtained results mainly provide atomic-scale insights into the local structural dynamics, coordination environments, and element interactions of the molten alloy. After the AIMD simulations, the equilibration snapshots were extracted from the trajectories and subsequently used for DFT calculations.

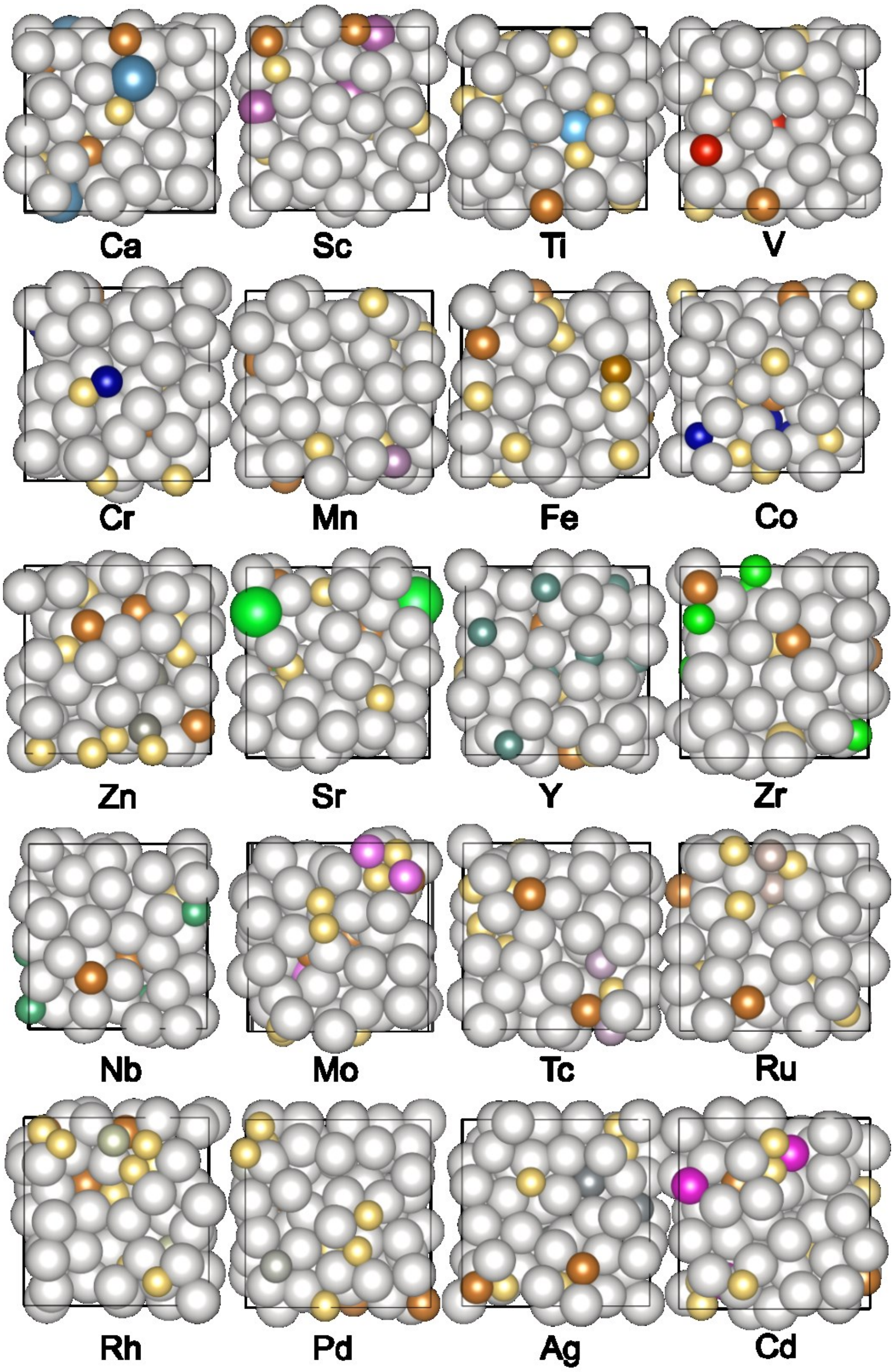

Figure S4. Representative snapshots of $Bi_{70}Ni_{18}Cu_6X_6$ (X = Ca, Sc, Zr, Ti, etc.) from AIMD simulations at 1500 K after structural equilibration for 10 ps.

Table S1. Average Bader charges of 20 elemental dopants in the $Bi_{70}Ni_{18}Cu_6X_6$ alloy calculated from AIMD simulations, with statistical averaging performed over the

trajectory starting from 1 ps.

Solute metal X	Bader charge	Solute metal X	Bader charge
Ca	-1.158	Y	-1.110
Sc	-1.081	Zr	-0.901
Ti	-0.821	Nb	-0.564
V	-0.499	Mo	-0.158
Cr	-0.219	Tc	0.120
Mn	-0.088	Ru	0.333
Fe	0.127	Rh	0.445
Co	0.153	Pd	0.364
Zn	-0.065	Ag	0.288
Sr	-1.206	Cd	-0.094

Table S2. The diffusion coefficients of the corresponding AIMD of the above-mentioned metal elements and the adsorption energy values when they are used as adsorption sites

Solute metal X	Diffusion coefficient	H* adsorption energy
Ca	3.215	-3.302
Sc	5.341	-2.037
Ti	2.463	-1.965
V	2.425	-2.239
Cr	2.958	-1.863
Mn	3.015	-2.528
Fe	6.265	-3.135
Co	6.865	-2.943
Zn	9.138	-2.583
Sr	2.388	-2.271
Y	1.709	-2.186
Zr	2.472	-2.024
Nb	5.023	-2.161
Mo	4.391	-2.215
Ru	3.984	-2.351
Rh	5.306	-2.542
Pd	6.921	-2.532
Ag	7.363	-1.965
Cd	8.602	-2.252

3. Experimental setup and validation

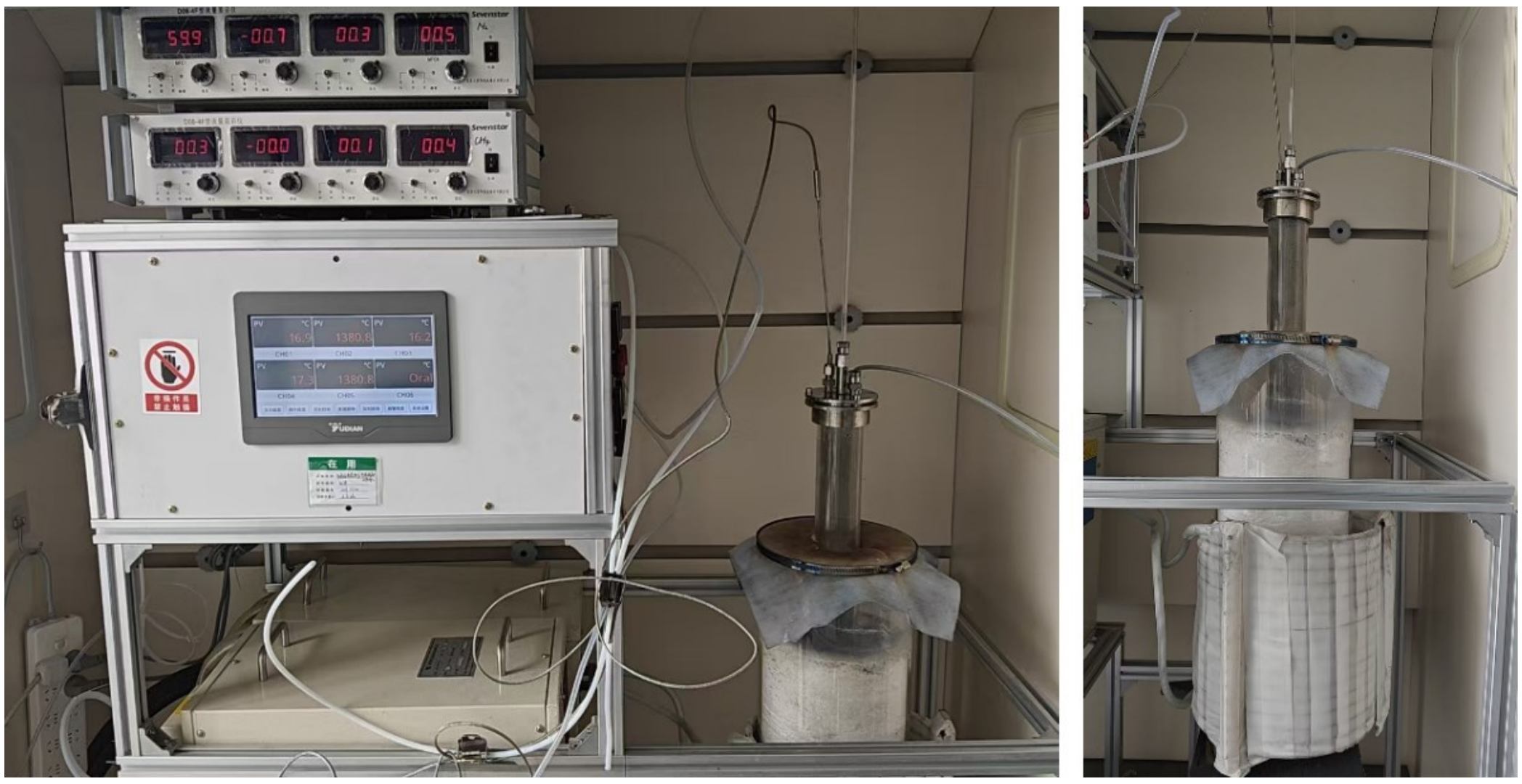

Figure S5. **Experimental Setup for Molten-Metal Bubble Catalysis and Final Experimental Conditions**. The experimental platform primarily consists of a high-frequency induction furnace, a cooling tower, a multi-channel bubble-melting reactor, and an external gas chromatograph for monitoring the hydrogen production rate.

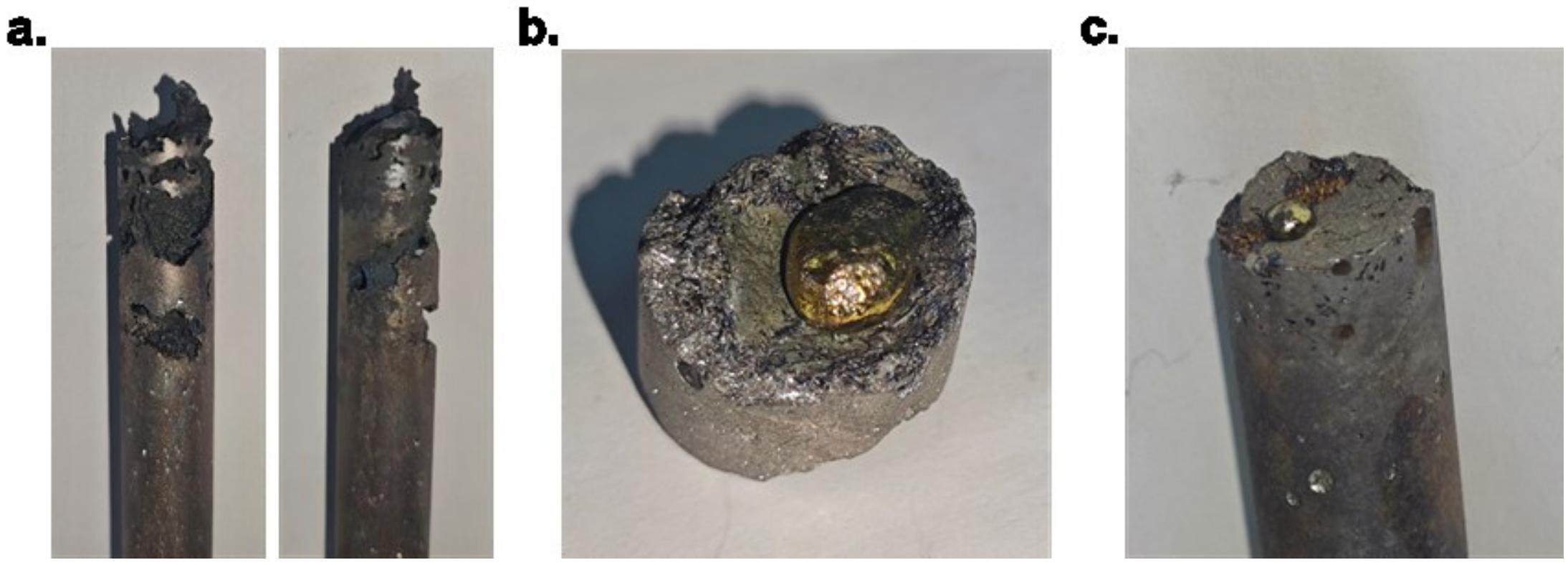

Figure S6. **Morphology of the Molten Metal Column after the Introduction of Fe.** It was observed that under identical heating and melting conditions, the Fe metal spheres did not fully dissolve into the molten alloy. Owing to the higher density of molten Bi, the undissolved Fe particles migrated from the bottom of the reactor to the liquid surface. (a) The metal column became obstructed by the undissolved Fe, and a large cavity formed upon cooling. (b)–(c) After fracturing the solidified metal column, several Fe spheres were found to remain undissolved within the alloy matrix.

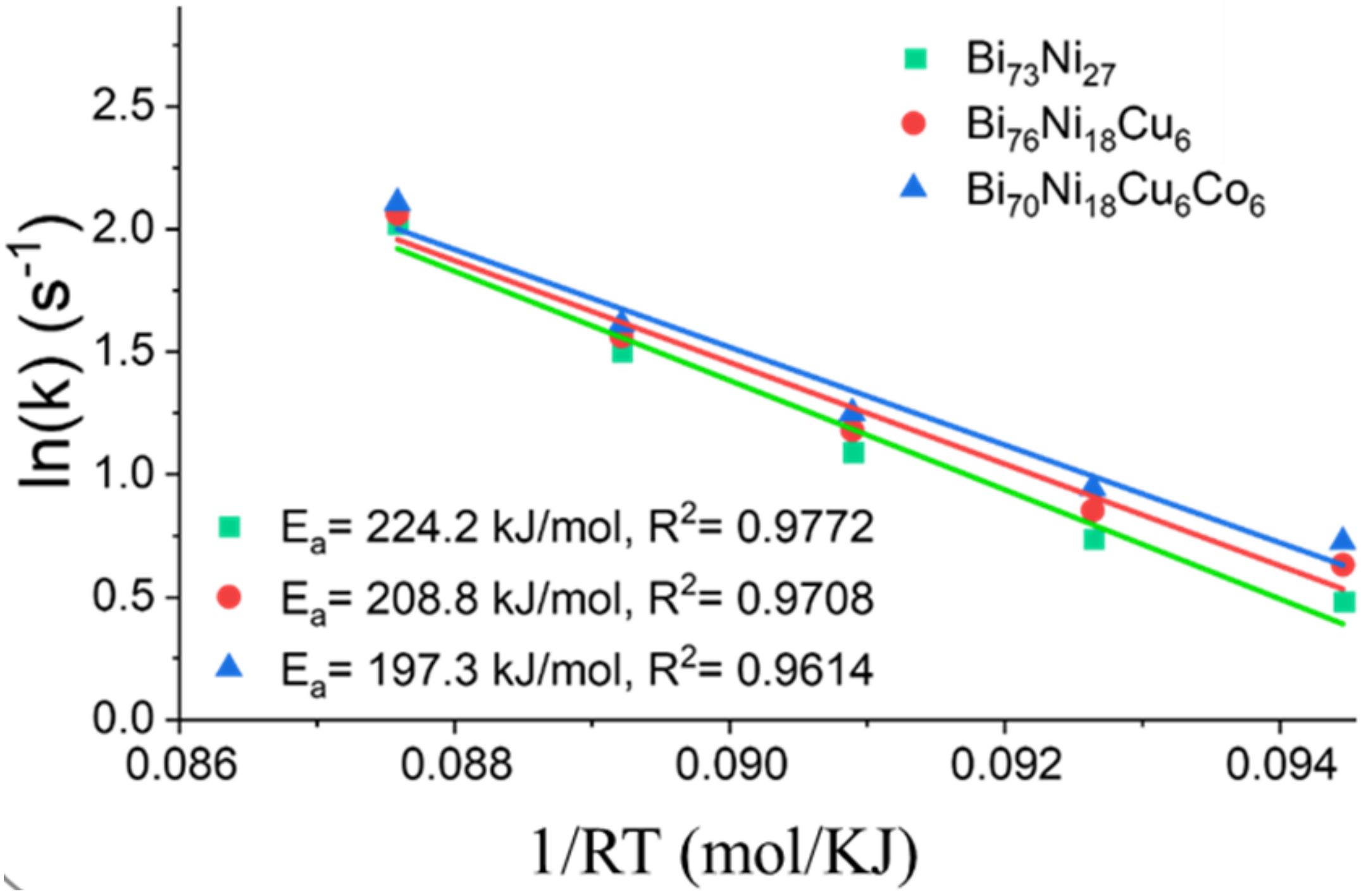

Figure S7. Arrhenius plot fitted using 1/RT as the x-axis for $Bi_{73}Ni_{27}$, $Bi_{76}Ni_{18}Cu_6$, and $Bi_{70}Ni_{18}Cu_6Co_6$ molten catalysts

4. The CFD simulation of bubble behavior

Molten metals are opaque, the bubble behavior in it is difficult to be observed. Therefore, Ansys Fluent was used to simulate gas liquid two phase flow inside the reactor and quantify bubble volume, formation/detachment period, residence time, and coalescence statistics. The VOF method was employed to track the gas/liquid interface. Pressure velocity coupling was solved using the COUPLED algorithm, and turbulence was modeled with the standard k-ε equations. The computational geometry matched the experimental dimensions, and the polyhedral mesh was used to discretize the computational domain with the mesh count of 130000 (Figure S8). The temperature-dependent densities, viscocities and surface tensions of the investigated alloys were estimated by linear interpolation between those of the corresponding pure metals at the process temperature [1], and presented in Table S3. The required thermophysical data for the pure components were taken from Smithells Metals Reference Book [2]. The

gas phase was methane (50%) and Argon (50%). Chemical reactions and heat transfer were neglected, however, because the gas expands at elevated temperature, methane inlet properties were set using the ideal-gas relation at 1000 °C. A contact angle of 130° was specified at the inlet quartz and ceramic surfaces to represent non-wetting behavior between the molten metal and quartz, ceramic materials. Bubble formation and detachment periods were extracted from the periodic pressure fluctuations at the inlet orifice, and bubble rise trajectories were used to obtain residence time and coalescence counts.

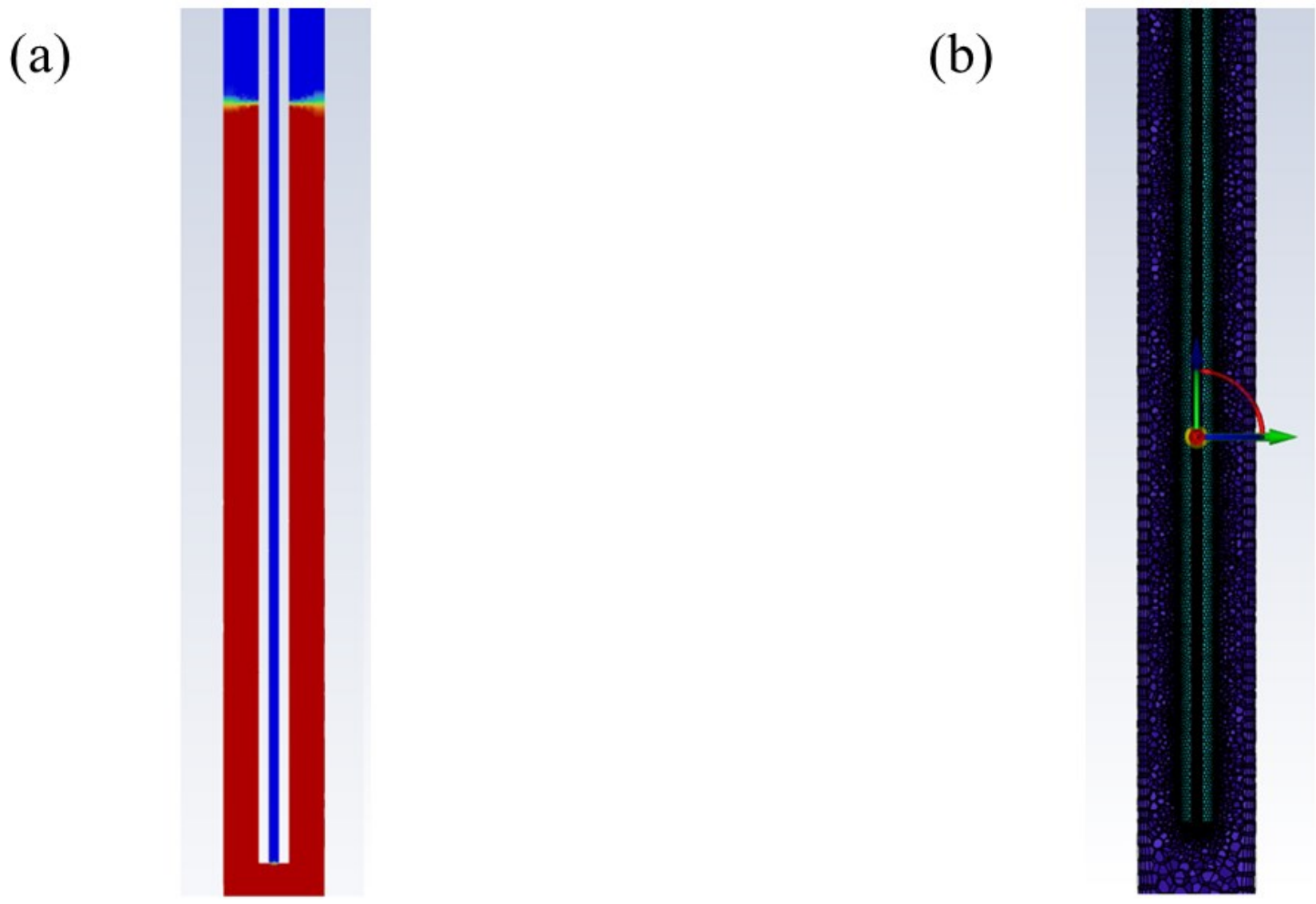

Figure S8. (a) Model geometry for CFD simulation; (b) Generated polyhedral mesh.

Table S3. Alloys (element contents in at%) and their parameter at 1000 °C

Alloys	Density [kg/m^3]	Viscosity [mPa·s]	Surface tension (mN/m)
$Bi_{73}Ni_{27}$	9120	1.36	460
$Bi_{76}Ni_{18}Cu_6$	9180	1.26	440
$Bi_{70}Ni_{18}Cu_6Co_6$	9150	1.32	560

References

[1] Scheiblehner D, Neuschitzer D, Wibner S, Sprung A, Antrekowitsch H. H ydrogen

production by methane pyrolysis in molten binary copper alloys. I nt J Hydrogen Energy. 2023;48(16):6233–6243. https://doi.org/10.1016/j.ijhy dene.2022.08.115.
[2] Totemeier TC, ed. Smithells Metals Reference Book. 8th ed. Amsterdam: Elsevier Butterworth-Heinemann; 2004.

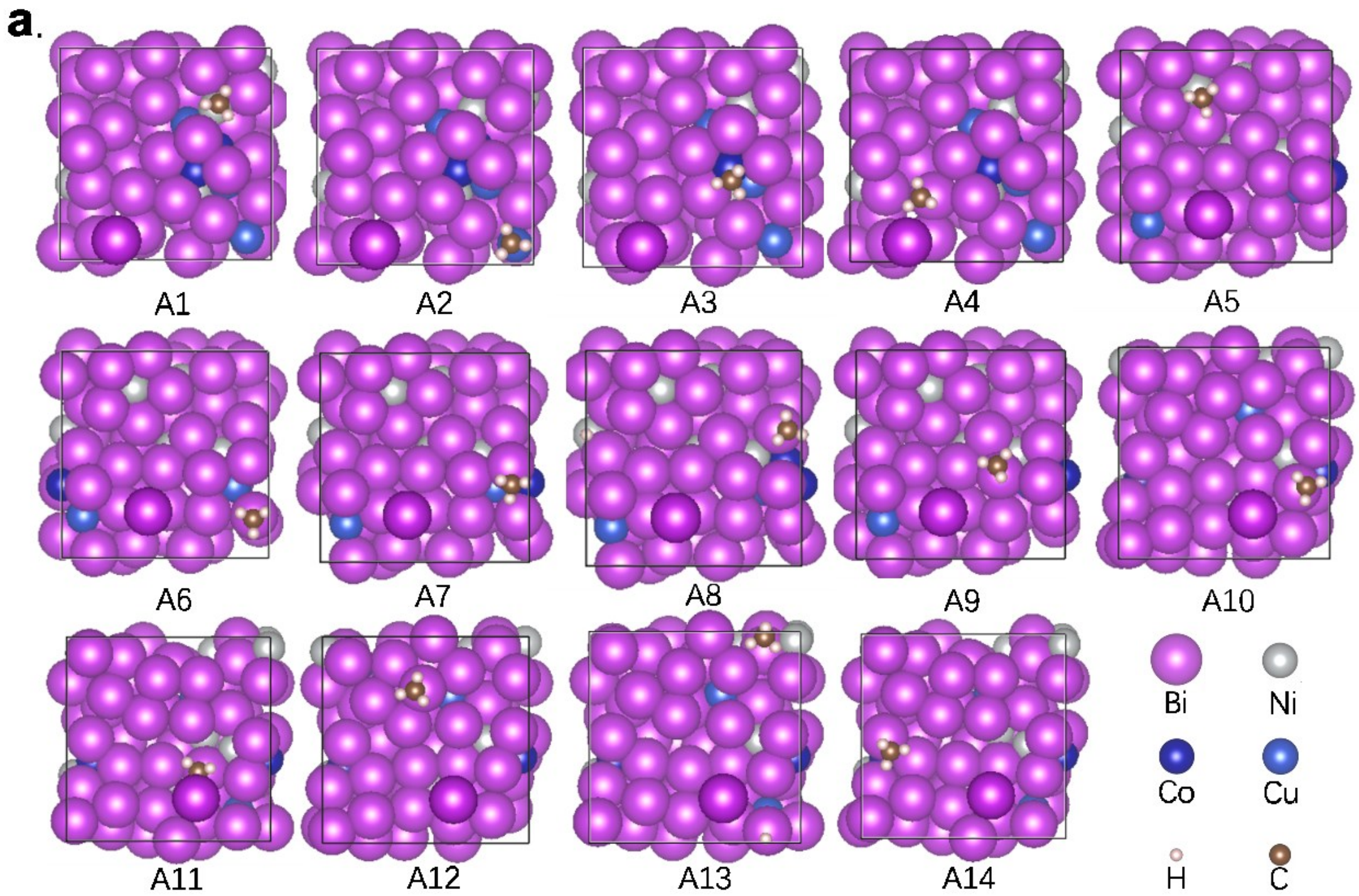

5. Microscopic catalytic simulation

Conf.	Index	Adsorption energy (eV)
	A1	-1.687
	A2	-2.26
	A3	-2.332
	A4	-2.102
	A5	-1.765
	A6	-2.672
CH_3*	A7	-2.106
	A8	-1.913

	A9	-1.844
	A10	-2.205
	A11	-1.905
	A12	-1.955
	A13	-2.387
	A14	-1.944

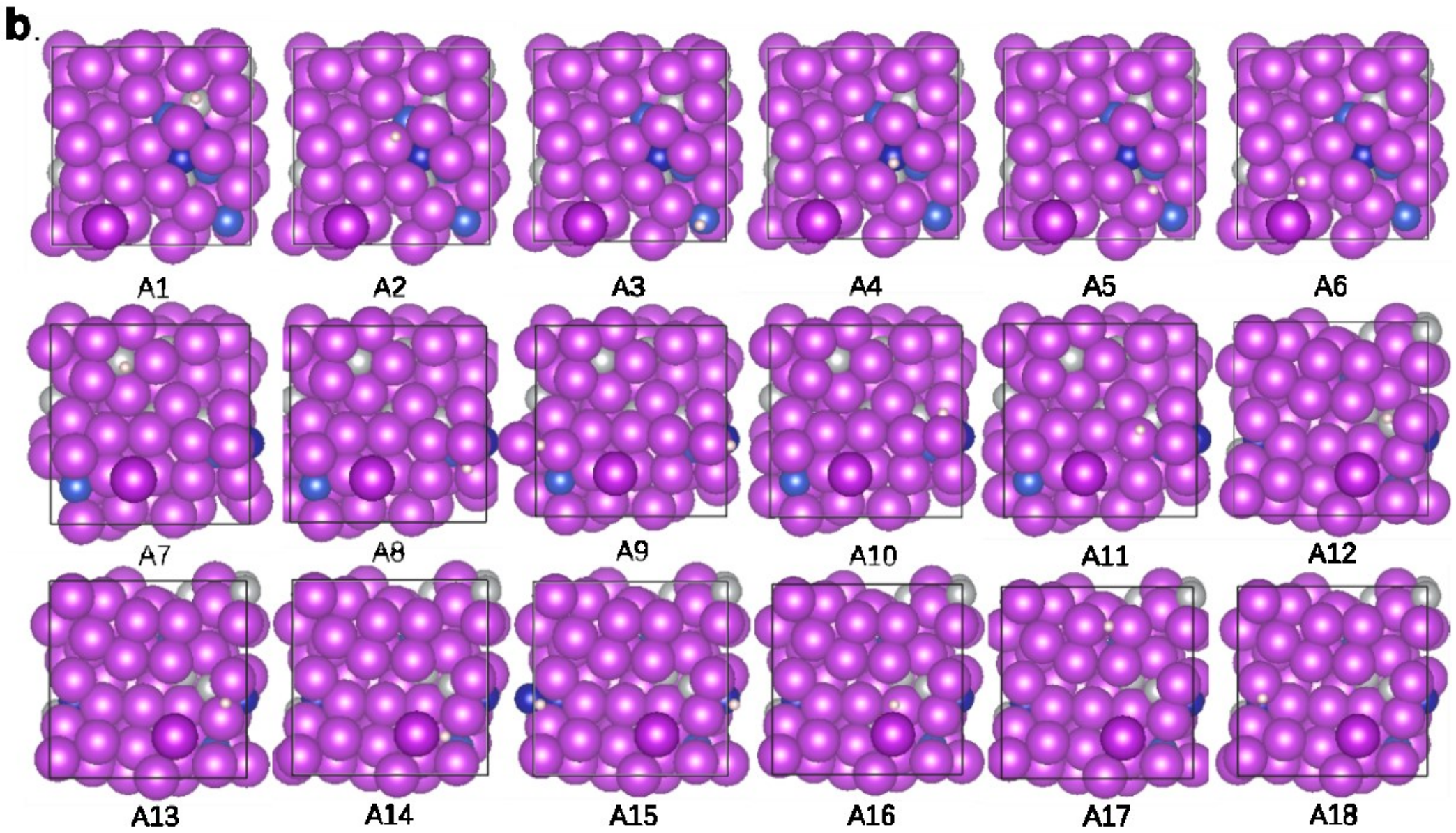

Conf.	Index	Adsorption energy (eV)
H*	A1	-2.060
	A2	-1.622
	A3	-1.934
	A4	-2.530
	A5	-1.570
	A6	-1.724
	A7	-1.904
	A8	-1.719
	A9	-1.788
	A10	-1.605
	A11	-1.490
	A12	-2.022

	A13	-1.790
	A14	-1.821
	A15	-2.176
	A16	-1.541
	A17	-1.677
	A18	-1.605

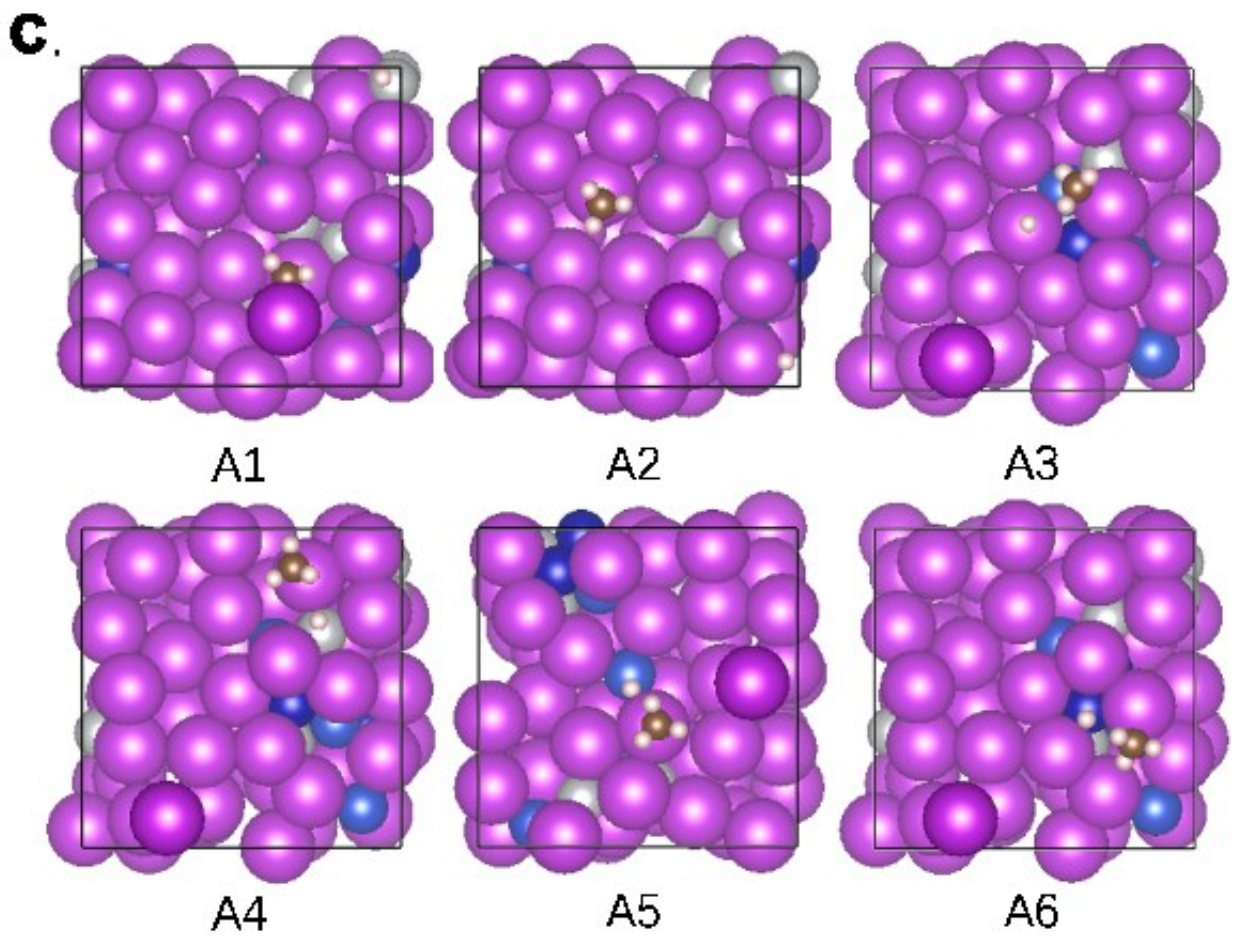

Conf.	Index	Adsorption energy (eV)
	A1	-1.649
	A2	-1.586
CH_3* +H*	A3	-2.170
	A4	-1.344
	A5	-1.856
	A6	-2.213

Figure S9. The DFT-optimized CH_3*, H*, CH_3*+H* adsorption configurations on different surface sites of the $Bi_{70}Ni_{18}Cu_6Co_6$ model and their corresponding adsorption energies are shown in the table.

Table S4. Comparison of metal diffusion coefficients in $Bi_{73}Ni_{27}$, $Bi_{76}Ni_{18}Cu_6$, and $Bi_{70}Ni_{18}Cu_6Co_6$.

Catalyst	Element	Diffusivity (10^{-9} m^2/s)
$Bi_{73}Ni_{27}$	Bi	4.585
	Ni	6.553
	The total	4.979
$Bi_{76}Ni_{18}Cu_6$	Bi	5.346
	Ni	7.908
	Cu	4.031
	The total	5.108
$Bi_{70}Ni_{18}Cu_6Co_6$	Bi	5.655
	Ni	6.091
	Cu	3.572
	Co	6.349
	The total	5.652

Table S5. Comparison of the Bader charge differences of surface metal atoms in various quaternary alloy systems before and after H adsorption, and their correlation with the corresponding adsorption energies.

Adsorption site	Bader charge (Before adsorption)	Bader charge (After adsorption)	Net charge transfer quantity	H* adsorption energy
Co	9.30347	9.28319	0.02029	-2.28909
	9.21125	9.18343	0.02782	-2.28824
	9.22604	9.14593	0.08011	-2.27339
Ni	10.24734	10.16932	0.04256	-2.06049
	10.20964	10.16919	0.04045	-2.02232
	10.28457	10.27721	0.00736	-1.90437
Cu	11.08963	11.01372	0.07591	-1.97665
	11.15373	11.04226	0.11147	-1.81473
	11.10151	10.98395	0.11757	-1.72474
Bi	14.95302	14.75782	0.19520	-1.57042
	14.88752	14.65739	0.23013	-1.54129
	14.90637	14.67265	0.23372	-1.49053

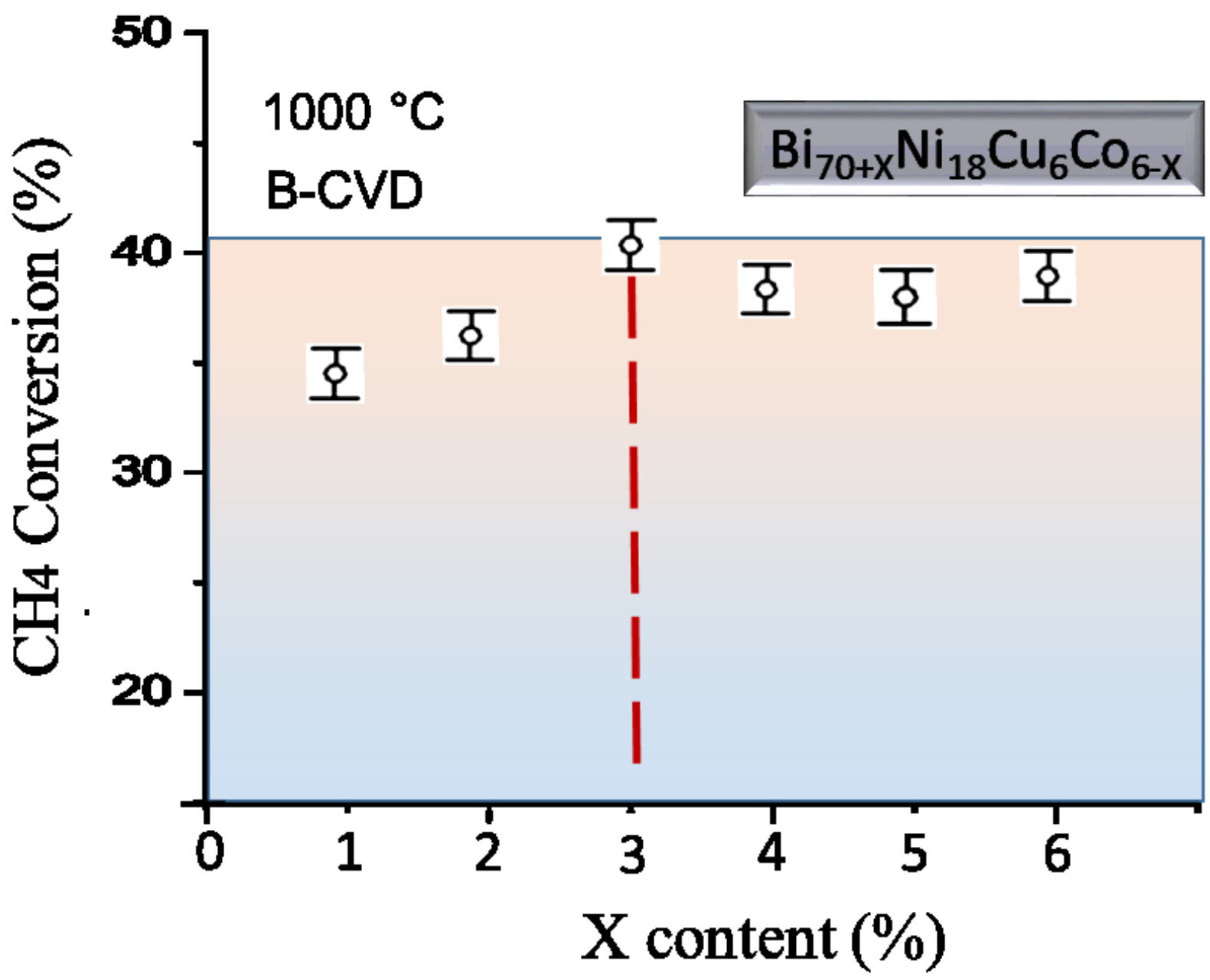

Figure S10. The experiment evaluated the CH_4 conversion efficiencies of $Bi_{70+X}Ni_{18}Cu_6Co_{6-X}$ by varying the Co content (X = 1% ~ 6%) at 1000 °C
